# Prevalence of target organ damage in rural Kenya and The Gambia: a cross-sectional study

**DOI:** 10.1016/j.lanafr.2026.100087

**Published:** 2026-09

**Authors:** Alexander D. Perkins, Modou Jobe, Ruth Lucinde, Juliet Otieno Awori, Sampa Sanneh, Catherine Kalu, Clement Mwagwabi, Elminah Saru, Gabriela M.M. Paixão, Kieran Mwazo, David A. Leon, Tim Clayton, Emily Herrett, Adrianna Murphy, Elijah Nyainda Ogola, Georg Ehret, Aletta E. Schutte, Andrew M. Prentice, Anoop S.V. Shah, Anthony Etyang, Pablo Perel, IHCoR-Africa Collaborative, Benjamin Tsofa, Edwine Barasa, Robinson Oyando, Samson Kinyanjui, Noni Mumba, Jemima Kamano, Violet Naanyu, Lilian Mbau, Nadia Aliyan, Nancy Kagwanja, James Abuje, Assan Jaye, Lamin Jobarteh, Saidina Ceesay, Ellen Nolte, Melanie Morris, David Prieto-Merino, Cova Bascaran, Syreen Hassan, Mavis Foster-Nyarko

**Affiliations:** a Department of Non-communicable Disease Epidemiology, London School of Hygiene & Tropical Medicine, London, UK; b Nutrition & Planetary Health Theme, Medical Research Council Unit The Gambia at LSHTM, Banjul, the Gambia; c Department of Epidemiology and Demography, KEMRI-Wellcome Trust Research Programme, Kilifi, Kenya; d Universidade Federal de Minas Gerais, Belo Horizonte, Brazil; e Coast General Teaching Hospital, Mombasa, Kenya; f Department of Medical Statistics, London School of Hygiene & Tropical Medicine, London, UK; g Department of Health Services Research and Policy, London School of Hygiene & Tropical Medicine, London, UK; h Department of Clinical Medicine and Therapeutics, University of Nairobi, Nairobi, Kenya; i Department of Cardiology, Geneva University Hospitals, Geneva, Switzerland; j School of Population Health, University of New South Wales, Kensington Campus, Sydney, NSW, Australia; k The George Institute for Global Health, Sydney, NSW, Australia; l Department of Cardiology, Imperial College Healthcare NHS Trust, London, UK

**Keywords:** Hypertension, Cardiovascular disease, Target organ damage, Hypertension-mediate organ damage, Sub-Saharan Africa

## Abstract

**Background:**

Sub-Saharan Africa is experiencing a hypertension crisis, potentially contributing to early development of target organ damage (TOD), a cardiovascular disease (CVD) predictor. However, comprehensive TOD prevalence data are lacking in rural SSA. This study aimed to provide the first population-level assessment of TOD prevalence in rural SSA.

**Methods:**

We conducted a cross-sectional, population-based study in Kilifi (Kenya) and Kiang West (The Gambia) from July 2023–December 2024. We assessed an age-stratified random sample of adults (≥30 years) for 24-h ambulatory blood pressure (BP), electrocardiography, echocardiography, pulse-wave velocity, and renal function. TOD was defined as left ventricular hypertrophy (LVH), reduced global longitudinal strain (GLS), left ventricular systolic dysfunction (LVSD), arterial stiffness, or renal dysfunction.

**Findings:**

Of 1819 screened, 1379 were recruited, and 1212 were analysed. The mean age was 56.8 ± 14.8 years, 62.9% were female, 33.5% had hypertension. Overall, 57.4% had TOD and prevalence increased with BP (non-elevated BP: 35.0%; hypertension: 77.3% for; p < 0.001) and age (30–39 years: 32.7%; ≥70 years: 77.0%; p < 0.001). TOD prevalence varied by marker: electrocardiogram LVH (31.1%), echocardiogram LVH (10.0%), GLS (16.7%), LVSD (9.8%), arterial stiffness (38.0%), and renal dysfunction (3.8%). Prevalence was higher in The Gambia (64.8%) than Kenya (50.9%) (p < 0.001). Clustering of TOD markers increased with BP.

**Interpretation:**

TOD is common in rural SSA. These findings provide important information to guide the implementation of TOD assessment in cardiovascular care pathways and inform the optimal ages for CVD screening.

**Funding:**

National Institute for Health and Care Research, Medical Research Council, and Wellcome Trust.

**Research in context**

**Evidence before this study**

We searched MEDLINE, EMBASE, the Web of Science Core Collection, and Global Health databases in October 2024 and updated the searches in October 2025. Structured and unstructured search terms captured hypertension, prevalence, target organ damage (TOD), cardiac TOD (left ventricular hypertrophy, left ventricular dysfunction, global longitudinal strain), vascular TOD (arterial stiffness), and renal TOD (renal dysfunction) including synonyms and alternative terminology. Searches had no time limits, included only primary research, and were restricted to African populations. Although several studies were identified, they predominantly involved urban, hypertensive, or healthcare-facility samples. We found no comprehensive, population-level assessments of TOD from rural settings.

**Added value of this study**

To our knowledge, this is the first study to provide a detailed population-level estimate of TOD prevalence in sub-Saharan Africa. Our study adds significant value in several ways. Using population registries, we recruited a random age-stratified sample of adults. We assessed TOD using reference-standard diagnostic tools coupled with detailed blood pressure measurements, generating a uniquely rich dataset. We analysed clustering of TOD markers and calculated age- and sex-standardised prevalence estimates to the WHO Standard Population to facilitate comparison with other regions.

**Implications of all the available evidence**

Our study advances the understanding of TOD epidemiology in sub-Saharan Africa by comprehensively characterising TOD in a randomly selected adult population. We demonstrate that TOD is common, including in younger adults and women, with greater clustering at higher blood pressure levels. Given uncertainties about the applicability of established cardiovascular risk scores in African populations, TOD provides a valuable alternative marker of risk. In this context, our findings have important implications for hypertension care in low-resource settings, indicating a need to consider how TOD assessment could be incorporated into cardiovascular care pathways and to refine age thresholds for screening. Although conventional TOD diagnostics are unrealistic for routine use in low-resource and rural settings, emerging point-of-care tools may offer opportunities. Validation and implementation studies are urgently needed.

## Introduction

In Africa, hypertension affects nearly one-in-two adults and is the leading cause of death among older adults.^1^^,^^2^ Compared to other parts of the world, hypertension in Africa presents earlier, is more severe, less controlled, and more likely to result in long-term cardiovascular disease (CVD) and premature mortality.^3^, ^4^, ^5^ Patients admitted with acute CVD in Africa are a decade younger compared to those in high-income countries (HIC) and experience three-fold higher case fatality rates.^6^^,^^7^

The clinical consequences of hypertension are concerning in rural Africa where a high prevalence of hypertension is compounded by low awareness, treatment and control.^8^ Despite the proven efficacy of antihypertensive pharmacotherapy, sustainable and effective hypertension management in these settings remains unachieved due to shortfalls in healthcare infrastructure, workforce, and essential medicine accessibility.^3^^,^^9^

These challenges contribute to the early onset and increased severity of target organ damage (TOD) a collective term for subclinical structural and functional changes to the heart, arteries, kidneys, brain, and eyes, caused by or associated with prolonged exposure to elevated blood pressure.^10^^,^^11^ Target organ damage is an early marker of cardiovascular sequelae and its assessment enhances risk stratification and management of hypertension.^12^ Presence of organ damage in the absence of hypertension is predictive of CVD.^13^

This study, part of the *Improving Hypertension Control in Rural Sub-Saharan Africa* (IHCoR-Africa) project, aimed to provide the first comprehensive assessment of population-level TOD prevalence in two rural African communities employing reference-standard imaging and diagnostic tools.^14^ Our study fills an important knowledge gap in a region where large-scale studies on the epidemiology of TOD remain scarce. Existing data are generally restricted to hypertensive populations; are based in urban or hospital settings; and do not use reference-standard diagnostic tools.^15^, ^16^, ^17^, ^18^

## Methods

### Study design and participants

The study design has been reported previously.^14^ It was conducted in Kilifi County, Kenya and Kiang West, The Gambia. Both regions are among the poorest in their respective countries, with estimates showing a high prevalence of hypertension but hypertension treatment rates less than 5%.^19^^,^^20^ Both sites are covered by Health and Demographic Surveillance Systems (HDSS), which include all residents.^21^^,^^22^ An age-stratified random sample of 1250 potential participants (625 in each country) aged ≥30 years in 10-year age-strata was identified from the HDSSs in both countries. Sample size justification is provided in the Supplementary Information. Study staff screened participants to confirm eligibility and request informed consent. Eligibility criteria were broad: ≥30 years of age and able to provide informed consent. People unable to perform any of the study procedures and pregnant women were ineligible. We have followed the STROBE guidelines for reporting cross-sectional studies.

### Study procedures

#### Demographics, medical history, anthropometrics, and blood biochemistry

Participants provided data on age, self-reported sex, employment status, tobacco use, and medical history. We measured height and weight and collected a venous blood sample. Ethnicity data were not collected.

#### Blood pressure measurement

All participants underwent 24-h ambulatory blood pressure measurement (24-h ABPM) using validated devices. Blood pressure was categorised using mean 24-h systolic and diastolic blood pressure (SBP and DBP) according to the 2024 European Society of Cardiology definitions (see Supplementary Information).^12^

#### Target organ damage assessment

We assessed the prevalence of six TOD markers in three organ systems: the heart, arteries, and kidneys. Cardiac TOD markers included left ventricular hypertrophy (LVH) by electrocardiograph using the Sokolow-Lyon, Cornell and Romhilt-Estes criteria (LVH-ECG), LVH by echocardiography using LV mass indexed to the allometric growth rate (LVH-Echo), reduced global longitudinal strain (GLS), and left ventricular systolic dysfunction (LVSD) based on LV ejection fraction. The arterial TOD marker was arterial stiffness based on aortic pulse wave velocity (aPWV). The renal TOD marker was renal dysfunction based on estimated glomerular filtration rate (eGFR). We also report LVH indexed to body surface area (LVH-BSA).

Full details of blood pressure and TOD measurement, assessment, and definitions, are provided in the Supplementary Information. Data collection procedures were standardised across both sites to maximise comparability.

### Statistical analysis

#### Prevalence analysis

The primary analysis calculated TOD prevalence for participants with at least one valid TOD assessment and at least one diurnal and one nocturnal 24-h ABPM reading. Sample characteristics were summarised using simple descriptive statistics with counts and percentages for categorical variables and means and standard deviation for continuous variables. Prevalence is reported for individual TOD markers stratified by blood pressure category, country, age, and sex. Age- and sex-standardised prevalences and means were calculated using the WHO 2000–2025 World Standard Population.

For composite analyses, any TOD was defined as the presence of at least one of LVH-ECG, LVH-Echo, reduced GLS, mildly reduced or reduced LVSD, arterial stiffness, or renal dysfunction. Composite analyses report prevalence with 95% confidence intervals calculated using the Wilson Score with continuity correction.

#### Blood pressure association with target organ damage

The association between blood pressure categories and TOD prevalence was analysed using the Chi-square test of independence, or Fisher's Exact test if expected frequency was <5. Differences in continuous measures across blood pressure categories were analysed using one-way analysis of variance.

#### Sensitivity analysis

We conducted a sensitivity analysis for population selection examining: all consented participants with at least one 24-h ABPM reading; the previously defined primary analysis population; and a population defined using the International Database of Ambulatory Blood Pressure in relation to Cardiovascular Outcome (IDACO) criteria for 24-h ABPM (≥70% successful readings, ≥10 diurnal and ≥5 nocturnal measurements). Baseline characteristics were compared. The association between TOD and blood pressure was re-analysed in the IDACO-criteria sample.

#### Clustering analysis by left ventricular hypertrophy or cardiac function

We evaluated the TOD clustering (the number of markers within an individual) as assessed by surface 12-lead ECG, echocardiography, and renal function. We derived composite cluster variables for the presence of one, two, three, four, or five TOD markers. Prevalence for each composite variable was stratified by blood pressure category. Given the high degree of missingness in PWV measurements in The Gambia, we did not include arterial stiffness in the clustering analysis.

The association between blood pressure category and TOD clustering was analysed using a Chi-square test of independence, or Fisher's Exact test if expected frequency was <5. To explore how TOD markers cluster, UpSet plots were produced showing the prevalence of all potential combinations of TOD clusters by blood pressure category.

All analyses were conducted using the R statistical software suite (R version 4.5.0).

### Ethical considerations and study registration

This study was carried out in accordance with International Council for Harmonisation–Good Clinical Practice and approved by ethics committees at KEMRI-Wellcome Trust Research Programme (SERU 4620), the Joint Gambia Government/MRCG Ethics Committee, and the London School of Hygiene and Tropical Medicine (LSHTM; LEO 28276). All participants provided written informed consent prior to enrolment. We facilitated access to care for participants who were identified as hypertensive during the study (see Supplementary Information for details). The study was prospectively registered on the ISRCTN public database (ISRCTN35951727).

### Role of the funding source

The funder had no role in the design, conduct, interpretation, or reporting of this research.

## Results

Recruitment and data collection took place between July 2023 to December 2024. Figure S1 shows the participant flow from recruitment to data analysis. 1212 participants were included in the primary analysis. There were 641 participants from Kenya and 571 from The Gambia. The population characteristics are shown in Table 1. The mean age was 56.8 ± 14.3 years, and 762 participants were female (62.9%). Of the 1212 participants included, 686 (56.6%) had elevated blood pressure and 406 (33.5%) had hypertension. Participants with hypertension were older (mean age 62.2 ± 14.2 years) than those without (mean age 54.8 ± 15.3 years) and more likely to be male. Those with non-elevated blood pressure were also more likely to be female (78.3%) compared to elevated blood pressure (63.3%) and hypertension (57.6%). Mean body mass index (BMI) for the population was 22.6 kg/m^2^ and increased with BP. Serum biochemistry and lipid levels were within expected ranges. Across the population, 140 participants (11.6%) reported tobacco use. Self-reported history of CVD, diabetes, kidney disease and other chronic conditions was less than 5% across all blood pressure categories with exception of high cholesterol in those with hypertension (14.8%). Population characteristics stratified by country, age, sex and systolic blood pressure strata are presented in Tables S1–S4.

**Table 1:** Participant characteristics stratified by blood pressure category for the primary analysis population.

	All Participants	Non-elevated blood pressure	Elevated blood pressure	Hypertension
All participants, n (%)	1212 (100.0)	120 (9.9)	686 (56.6)	406 (33.5)
Demographics and anthropometrics				
Age in years, mean (SD)	56.8 (14.8)	54.8 (15.3)	54.1 (14.2)	62.2 (14.2)
Female, n (%)	762 (62.9)	94 (78.3)	434 (63.3)	234 (57.6)
Country	–	–	–	–
Kenya, n (%)	641 (52.9)	89 (74.2)	363 (52.9)	641 (52.9)
The Gambia, n (%)	571 (47.1)	31 (25.8)	323 (47.1)	217 (53.4)
Body mass index in kg/m^2^, mean (SD)	22.6 (4.4)	20.9 (4.0)	22.3 (4.2)	23.5 (4.8)
Formal schooling in years^@^, mean (SD)^@^	4.6 (4.7)	4.0 (4.0)	4.9 (4.7)	4.2 (4.7)
In employment, n (%)^@^	1034 (86.1)	102 (85.0)	611 (89.6)	321 (80.5)
Haemodynamics (24-h ABPM)				
Heart rate in bpm, mean (SD)	73.1 (9.4)	69.5 (9.3)	73.5 (9.2)	73.5 (9.5)
Systolic blood pressure in mmHg, mean (SD)	121.0 (18.1)	101.7 (7.2)	113.0 (8.0)	140.3 (16.3)
Diastolic blood pressure in mmHg, mean (SD)	74.3 (9.2)	61.3 (2.5)	71.2 (4.2)	83.3 (8.7)
Blood biochemistry and lipids				
Serum potassium in mmol/L, mean (SD)	4.1 (0.8)	4.2 (0.6)	4.1 (0.8)	4.0 (0.8)
Serum sodium in mmol/L, mean (SD)	139.5 (6.2)	138.4 (7.3)	139.7 (6.3)	139.5 (5.7)
Serum cholesterol in mmol/L, mean (SD)	4.5 (1.1)	4.4 (1.0)	4.5 (1.1)	4.7 (1.1)
Serum triglycerides in mmol/L, mean (SD)	1.0 (0.5)	1.0 (0.4)	0.9 (0.5)	1.1 (0.7)
Serum LDL cholesterol in mmol/L, mean (SD)	2.7 (0.9)	2.6 (0.8)	2.7 (1.0)	2.9 (0.9)
Serum creatinine in μmol/L, mean (SD)	69.5 (30.2)	66.0 (17.2)	67.9 (25.9)	73.2 (38.8)
Medical history^@^				
Tobacco use, n (%)	140 (11.6)	14 (11.7)	76 (11.1)	50 (12.3)
Previous cardiovascular disease, n (%)	43 (3.6)	4 (3.3)	24 (3.5)	15 (3.7)
Heart disease, n (%)	9 (0.7)	2 (1.7)	6 (0.9)	1 (0.2)
Heart attack, n (%)	15 (1.3)	1 (0.9)	9 (1.3)	5 (1.3)
Stroke, n (%)	21 (1.7)	1 (0.8)	11 (1.6)	9 (2.2)
Diabetes, n (%)	26 (2.2)	0 (0.0)	12 (1.8)	14 (3.5)
Kidney disease, n (%)	11 (0.9)	2 (1.7)	5 (0.7)	4 (1.0)
High cholesterol, n (%)	96 (8.0)	3 (2.5)	33 (4.9)	60 (14.8)
Asthma, n (%)	47 (3.9)	4 (3.3)	30 (4.4)	13 (3.2)
Tuberculosis, n (%)	18 (1.5)	2 (1.7)	10 (1.5)	6 (1.5)
Arthritis, n (%)	29 (2.4)	2 (1.7)	15 (2.2)	12 (3.0)
HIV, n (%)	26 (2.1)	2 (1.7)	17 (2.5)	7 (1.7)
Other chronic conditions, n (%)	15 (1.2)	3 (2.5)	8 (1.2)	4 (1.0)

Blood pressure categories defined using the 2024 European Society of Cardiology categories based on 24-h ambulatory blood pressure measurement. Categorical data reported as counts and percentages. Continuous data reported as mean and standard deviation. @ indicates self-reported data.

**Fig. 1:**
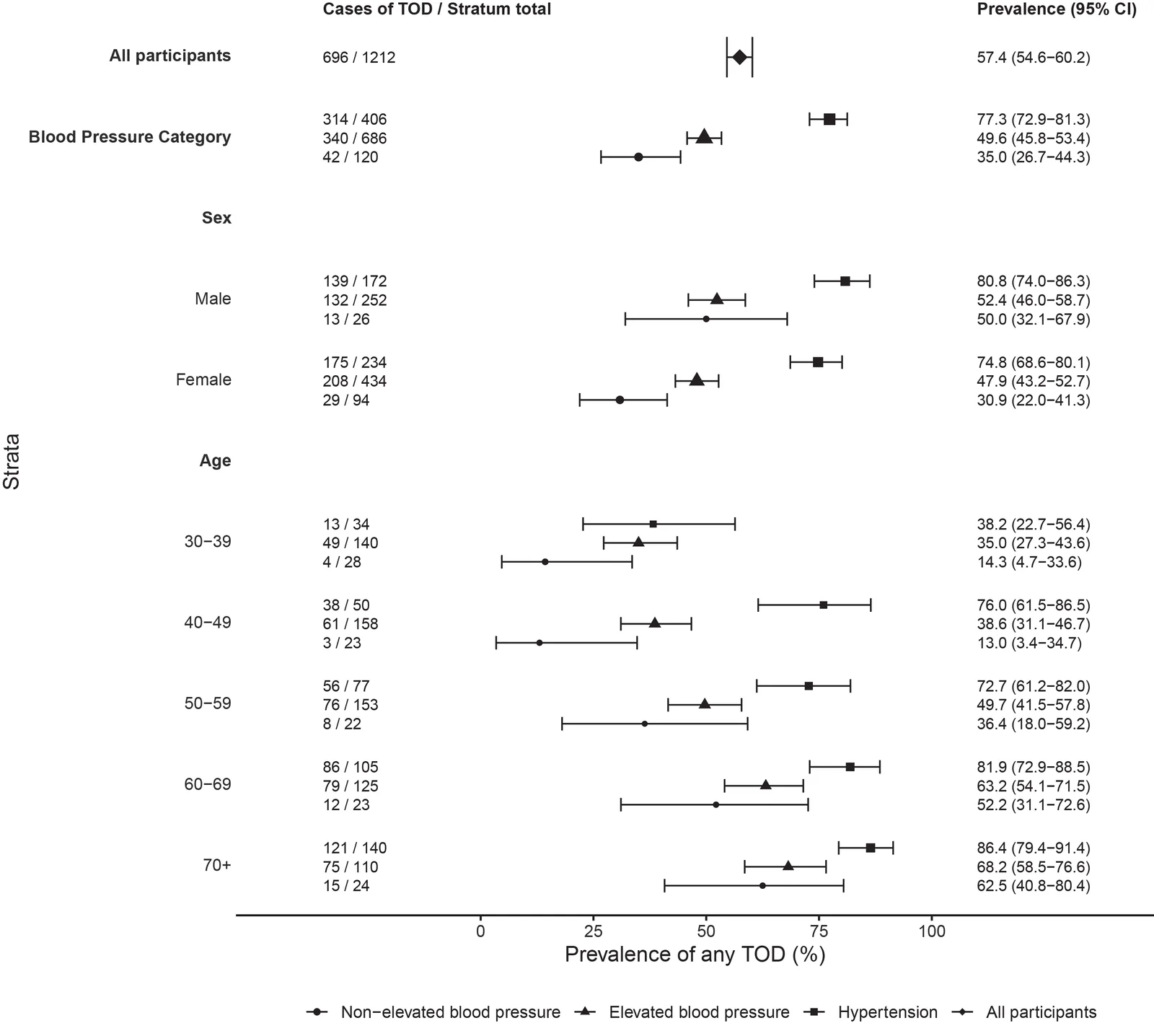
Forest plot of target organ damage prevalence stratified by sex and age, across blood pressure categories. Any TOD defined as presence of at least one marker of TOD. Blood pressure categories defined using the 2024 European Society of Cardiology categories based on 24-h ambulatory blood pressure measurement. CI is Confidence Interval.

Overall, 689 participants (57.4%) had at least one form of TOD (Table 2). When stratified by blood pressure category, the presence of any TOD showed a positive relationship with increasing blood pressure (35.0% for non-elevated blood pressure, 49.6% for elevated blood pressure, and 77.3% for hypertension, p < 0.001). This trend was maintained when stratified by country, sex, age, BMI (Fig. 1 and Figure S2).

**Table 2:** Prevalence of target organ damage stratified by blood pressure category for the primary analysis population.

	Overall	Non-elevated blood pressure	Elevated blood pressure	Hypertension	p-value
	1212	120	686	406	–
LVH-ECG, n (%)					
Absent	799 (68.9)	102 (87.2)	491 (74.8)	206 (53.4)	–
All LVH-ECG	360 (31.1)	15 (12.8)	165 (25.2)	180 (46.6)	<0.001
LVH-ECG without strain	297 (25.6)	13 (11.1)	146 (22.3)	138 (35.8)	–
LVH-ECG with strain	63 (5.4)	2 (1.7)	19 (2.9)	42 (10.9)	–
Missing	53	3	30	20	–
LVH-Echo, n (%)	115 (10.0)	7 (6.2)	39 (5.9)	69 (17.9)	<0.001
Missing	57	8	29	20	–
Global Longitudinal Strain, n (%)					
Normal	662 (59.6)	92 (82.9)	424 (67.3)	146 (39.5)	–
Borderline	263 (23.7)	15 (13.5)	141 (22.4)	107 (28.9)	–
Reduced	186 (16.7)	4 (3.6)	65 (10.3)	117 (31.6)	<0.001
Missing	101	9	56	36	–
LV Systolic Dysfunction, n (%)					
Normal	999 (90.2)	112 (99.1)	586 (93.0)	301 (82.5)	–
Mildly Reduced or Reduced	109 (9.8)	1 (0.9)	44 (7.0)	64 (17.5)	<0.001
Missing	104	7	56	41	–
Arterial Stiffness (n, %)	369 (38.0)	25 (23.6)	177 (33.3)	167 (49.9)	<0.001
Missing	240	14	155	71	–
Renal impairment (n, %)	44 (3.8)	3 (2.6)	16 (2.5)	25 (6.6)	0.003
Missing	66	6	33	27	–
Any TOD, n (%)	696 (57.4)	42 (35.0)	340 (49.6)	314 (77.3)	<0.001

For all prevalence estimates, the denominator is participants with an available measurement for the relevant target organ damage marker, excluding missing data. p-values are Chi-square test of independence, or Fisher's Exact test if expected frequency was less than 5, between the stratified groups. Blood pressure categories defined using the 2024 European Society of Cardiology categories based on 24-h ambulatory blood pressure measurement.

Prevalence of TOD was higher in The Gambia (64.8%) compared to Kenya (50.9%) (Table S5). Men (63.1%) had more TOD than women (54.1%) (Table S6). Notably, TOD prevalence remained high at younger ages (32.7% in the 30–39 year age strata) and increased with age (77.0% in the ≥70 year age strata) (Table S7). Prevalence of TOD increased across 20 mmHg strata of SBP (Table S8) These patterns were preserved across all individual TOD markers (Tables S5–S8). For continuous TOD variables, cardiac, arterial, and renal health were better in Kenya compared to The Gambia, in women compared to men, in younger people compared to older people, and in those with lower SBP (Tables S10–S13).

There was strong statistical evidence of an association between LVH-ECG prevalence and blood pressure category (Table 2, 12.8% for non-elevated blood pressure, 25.2% for elevated blood pressure, and 46.6% for hypertension, p < 0.001). The overall prevalence of LVH-ECG was 31.1%. LVH-ECG without strain was more prevalent than LVH-ECG with strain.

There was strong statistical evidence of an association between the prevalence of LVH-Echo and blood pressure category (6.2% for non-elevated blood pressure, 5.9% for elevated blood pressure, and 17.9% for hypertension, p < 0.001) (Table 2). The overall prevalence of LVH-Echo was 10.0%. When assessed as a continuous variable, there was strong statistical evidence of an association between LVMI to the allometric growth ratio and blood pressure and age, but not sex (Tables S9–S13).

LVH-Echo prevalence diverged significantly between countries (Table S5, overall: 10.0%, Kenya: 16.6%, The Gambia: 2.2%, p < 0.001). This was also the case for LVH-BSA (Table S14 Overall: 10.4%, Kenya: 17.0%, The Gambia: 2.8%, p < 0.001). LV mass was lower in The Gambia (110.0 g) than in Kenya (126.2 g) while BSA was higher in The Gambia (1.7 m^2^) compared to Kenya (1.5 m^2^). As a result, BSA indexed LV mass was lower in The Gambia (66.1 g/m^2^) compared to Kenya (81.2 g/m^2^). Unindexed LVH-IVSd prevalence was comparable in both settings (Overall: 12.7%, Kenya: 12.6%, The Gambia: 12.9%).

There was strong statistical evidence of an association between the prevalence of reduced GLS and blood pressure category (3.6% for non-elevated blood pressure, 10.3% for elevated blood pressure, and 31.6% for hypertension, p < 0.001) (Table 2). The overall prevalence of reduced GLS was 16.7%. In addition, there was strong statistical evidence of an association between GLS as a continuous variable and blood pressure category (p < 0.001) (Table S9).

There was strong statistical evidence of an association between the prevalence of LVSD and blood pressure category (0.9% for non-elevated blood pressure, 7.0% for elevated blood pressure, and 17.5% for hypertension, p < 0.001). The overall prevalence of LVSD was 9.8%. There was also strong statistical evidence of an association between LVEF as a continuous variable and blood pressure category (p < 0.001; Table S9).

Clinically-defined arterial stiffness was highly prevalent (38.0%). There was strong statistical evidence of an association between arterial stiffness prevalence and blood pressure category (23.6% for non-elevated blood pressure, 33.3% for elevated blood pressure, and 54.9% for hypertension, p < 0.001). There was strong statistical evidence of an association between PWV as a continuous variable and blood pressure category (p < 0.001; Table S9). Overall, 240 participants in the primary analysis did not have PWV data, and missingness showed no pattern across blood pressure categories (Table 2) although there was substantially more missing data in The Gambia compared to Kenya (204 vs 36; Table S5).

There was strong statistical evidence of an association between renal dysfunction prevalence and blood pressure category (Table 2, 2.6% for non-elevated blood pressure, 2.5% for elevated blood pressure, and 6.6% for hypertension, p = 0.003). The overall prevalence of renal dysfunction was 3.8%. There was also strong statistical evidence of an association between eGFR and blood pressure category (p < 0.001; Table S9).

There were no meaningful differences among the three populations included in the sensitivity analysis (Table S15). Compared to the primary analysis population participants in the stricter IDACO population were slightly younger (mean age 57.0 vs 56.8), and had almost identical blood pressure (121.0/74.3 mmHg vs 121.0/74.2 mmHg). The prevalence of any TOD in the IDACO-criteria population was slightly lower than the primary analysis population (57.4% vs 57.0%). The strong statistical evidence for the association between TOD and blood pressure category was preserved in the stricter sample for all individual and composite markers (Tables S16 and S17).

Age- and sex-standardising to the WHO Standardised Population Structure 2000–2025 which reduced the overall standardised prevalence of TOD to 50.0% (Table S18). This trend was observed across all individual TOD markers. Continuous TOD measures were also standardised and showed a shift towards reduced TOD. When stratified by blood pressure category the positive association between TOD prevalence and continuous measures, was preserved for all variables (Table S19). Standardised prevalence estimates and continuous measures were also stratified by country. In line with the unstandardised data, TOD burden was higher in The Gambia compared to Kenya (standardised prevalence of any TOD 56.3% and 44.2% respectively; Tables S20 and S21).

**Fig. 2:**
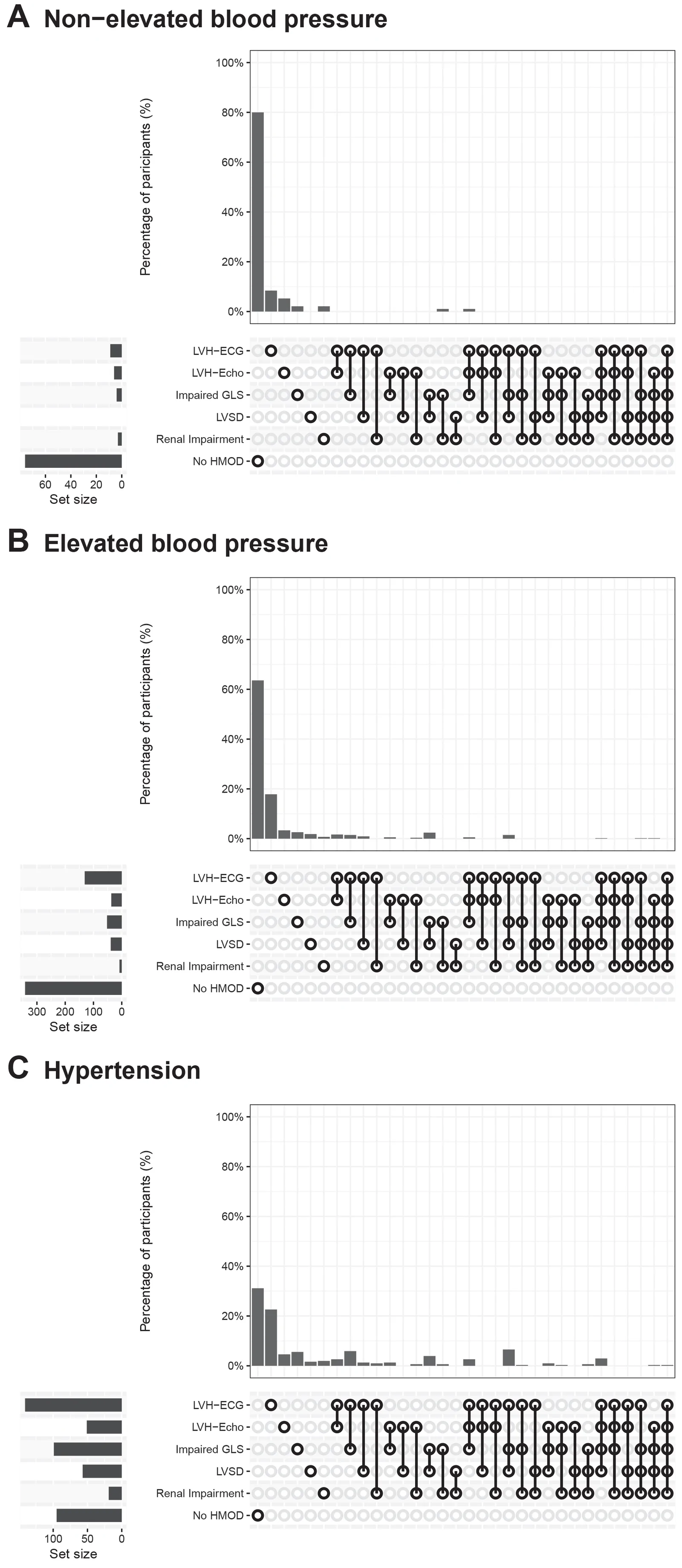
Clustering of target organ damage across blood pressure categories for cardiac and renal target organ damage markers. Panel A shows target organ damage clustering in participants with non-elevated blood pressure. Panel B shows target organ damage clustering in participants with elevated blood pressure. Panel C shows target organ damage clustering in participants with hypertension. All potential clusters are shown in the matrices as linked dots. Prevalence of each cluster is shown in the bar graphs. Set size shows the total number of participants with that marker. Target organ damage as defined elsewhere in the paper. Blood pressure categories defined using the 2024 European Society of Cardiology categories based on 24-h ambulatory blood pressure measurement.

There was strong statistical evidence that clustering of cardiac and renal TOD markers was associated with blood pressure categories across all combined markers of 2, 3, 4 or 5 forms of TOD (Table 3 and Fig. 2). For example, among those with non-elevated blood pressure, no participants had all four TOD markers. This increased to 3 participants (0.6%) among those with elevated blood pressure and 10 (3.3%) among those with hypertension (p < 0.001). There was also strong statistical evidence for clusters of two or three TOD markers. The prevalence of all potential combinations of TOD clusters increased by blood pressure category (Fig. 2). The cluster profile for all participants is shown in Figure S3. Clustering was higher in The Gambia compared to Kenya (Table S22).

**Table 3:** Prevalence of target organ damage clusters stratified by blood pressure category for cardiac and renal target organ damage markers.

	Overall	Non-elevated blood pressure	Elevated blood pressure	Hypertension	p-value
All participants, n (%)	938	95	538	305	–
No markers of TOD, n (%)	513 (54.7)	76 (80.0)	342 (63.6)	95 (31.1)	<0.001
One marker of TOD, n (%)	270 (28.8)	17 (17.9)	142 (26.4)	111 (36.4)	<0.001
Two markers of TOD, n (%)	94 (10.0)	1 (1.1)	40 (7.4)	53 (17.4)	<0.001
Three markers of TOD, n (%)	47 (5.0)	1 (1.1)	11 (2.0)	35 (11.5)	<0.001
Four markers of TOD, n (%)	13 (1.4)	0 (0.0)	3 (0.6)	10 (3.3)	0.002
Five markers of TOD, n (%)	1 (0.1)	0 (0.0)	0 (0.0)	1 (0.3)	0.354

p-values are Chi-square test of independence, or Fisher's Exact test if expected frequency was less than 5, between the stratified groups. Blood pressure categories defined using the 2024 European Society of Cardiology categories based on 24-h ambulatory blood pressure measurement.

## Discussion

Our study is an important advancement in the understanding of TOD epidemiology in rural Africa. We employed reference-standard diagnostic tools to deeply phenotype a randomly selected rural population. We report three key findings. First, the overall prevalence of TOD is very high in the rural African general adult population, including in younger people. Second, TOD prevalence increases from 3-in-10 for those with non-elevated blood pressure to 8-in-10 for those with hypertension. Third, TOD clustering increased with blood pressure category. These findings have important implications for hypertension care in the region.

To our knowledge, no studies have comprehensively evaluated TOD in the African general population, whether in rural or urban settings. Our study found a prevalence of TOD in over half the population, affecting nearly 6 out of 10 individuals. Given the absence, or relatively low levels, of traditional TOD risk factors such as obesity, tobacco use, diabetes and older age, the high prevalence of TOD in our study is striking. In line with previous evidence, TOD prevalence is higher in men than in women. Notably, the prevalence rates among younger adults in rural Africa found in our study mirrors that observed in significantly older individuals from the Framingham Heart Study cohort in the United States, suggesting that TOD manifests approximately a decade earlier in Africa.^13^ Importantly, other studies in Africa have been small, or have only evaluated TOD in hypertensive populations leaving a key gap in our understanding of the overall population prevalence.

Ours is the first study in Africa to demonstrate that TOD prevalence rises significantly across individuals according to blood pressure category, in line with evidence from HICs.^13^ We showed a high prevalence of subclinical cardiovascular damage across both countries and in both men and women. This observation likely reflects the high rates of undiagnosed and unmanaged hypertension across SSA.^3^ If unaddressed, the burden of CVD in the region will continue to increase. Of all cardiovascular pathologies, treating high blood pressure provides the greatest reduction in heart failure incidence, one of the most common forms of acute CVD in Africa. More concerning is that patients with heart failure in Africa present nearly a decade younger compared to their counterparts in the global North.^5^ We observed that 3-in-10 people with non-elevated blood pressure still had evidence of TOD, predominantly due to electrocardiographic LVH. This could be due to causes other than elevated blood pressure; misclassification of blood pressure; or misclassification of TOD. The accuracy of standard electrocardiographic LVH criteria in African populations remains controversial.^23^ However, even those without elevated blood pressure, presence of organ damage, including electrocardiographic LVH, is still a predictor of CVD risk.^13^

Among individual markers, the key drivers of overall TOD prevalence were left ventricular hypertrophy determined by surface ECG, arterial stiffness, and reduced cardiac function determined by GLS. Renal dysfunction was found in 1-in-25 participants and was consistent with other population-level reports in SSA.^24^ Left ventricular hypertrophy on ECG was more than double the prevalence observed in a hypertensive population in Sierra Leone and similar to prevalences observed in hypertensive populations in Ghana and Nigeria.^15^^,^^25^^,^^26^ The observed prevalence of LVH by electrocardiography was approximately three-fold that of LVH by echocardiography. Our study is the first population-level assessment of longitudinal cardiac function using speckle tracking in Africa, which is less susceptible to misclassification and provides early and independent prognostic information for cardiovascular disease.^27^ Our results underscore the hidden burden of TOD and the future potential burden of CVD.

Importantly, we observed a high prevalence of TOD clustering. Clustering analysis was limited to participants with cardiac and renal TOD data. Clustering of TOD was strongly associated with increasing blood pressure category. No participants with non-elevated blood pressure had clustering of four or five TOD markers, but among hypertensives, 1-in-10 had three markers, and 10-in-30 had all four and the only participant to have all five markers had hypertension. This high degree of clustering has important implications for the overall CVD risk in the population as TOD clustering leads to higher levels of long-term risk compared to the presence of a single marker.^13^^,^^28^ Our results are the first analysis of TOD clustering at a population-level in Africa and align with smaller studies in Africa and previous reports in HIC.^13^^,^^26^ The relationship between TOD clustering and long-term CVD outcomes in Africa has not been studied, and requires further investigation.

This study has four key strengths. First, it includes a randomly sampled population drawn from demographic surveillance systems which minimises selection bias. A small proportion of potential participants declined involvement but they were replaced with age- and sex-matched participants. Second, the study population was age-stratified to ensure a sufficient sample size to assess TOD prevalence in each age-strata. Third, TOD was deeply phenotyped using highly accurate diagnostics tools including advanced echocardiography which enabled the first population based assessment of GLS in Africa. Finally, TOD assessment was coupled with reference-standard blood pressure measurements using 24-h ABPM to produce a unique and highly detailed dataset for the exploration of TOD prevalence its relationship with blood pressure.

There are some limitations to this work. All TOD definitions were drawn from HICs and may not be applicable to SSA. The potential unsuitability of HIC-derived thresholds was evident in the divergent classification of LV mass indexed echocardiographic LVH. We acknowledge substantial missingness in PWV data in The Gambia due to challenges with devices. We do not have data on hypertension treatment rates prior to BP measurement in the IHCoR-Africa study. However, previous studies found a treatment rate under 5% in both settings suggesting that treatment is unlikely to impact TOD prevalence.^19^^,^^20^ As a cross-sectional study, we could not assess how TOD changes over time, or link long-term CVD outcomes to TOD. Future studies linking TOD in Africa with CVD outcomes and biomarkers, such as high-sensitivity troponin, are vital.

Our findings have important clinical and health policy implications for low-resource settings. We report on the epidemiology of TOD in a general adult rural African population and provide critical information for clinical guidelines, clinicians, and health policymakers to consider how TOD assessment should be embedded in the cardiovascular care pathway. We show that TOD is highly prevalent in younger populations and women which will inform the age cut-offs at which clinical screening for TOD should occur. Understanding the epidemiology of TOD is relevant in low-resource settings where the applicability of established cardiovascular risk estimation tools remains uncertain.^29^ Indeed, the latest clinical guidelines mandate the assessment of TOD for the management of hypertension.^11^^,^^12^ We acknowledge that it is not yet feasible, or realistic to propose, that TOD assessment be incorporated into routine care in rural Africa.^30^ However, novel point-of-care diagnostics, potentially supported by artificial intelligence, have the potential to increase access to community-based cardiovascular risk stratification. There is an urgent need for the validation and implementation of such technologies in Africa and other low resource settings.

## Contributors

Contributions follow the CReDIT taxonomy.

ADP: Conceptualisation, data curation, formal analysis, funding acquisition, investigation, methodology, project administration, visualization, writing—original draft, writing—reviewing and editing. MJ: Conceptualisation, data curation, funding acquisition, investigation, methodology, project administration, writing—reviewing and editing. RL: Investigation, methodology, project administration, writing—reviewing and editing. JOA: Investigation, methodology, project administration, writing—reviewing and editing. SS: Investigation and data curation. CK: Investigation and data curation. CM: Investigation and data curation. ES: Investigation and data curation. GMMP: Resources, data curation and writing—reviewing and editing. KM: Resources and investigation. DAL: Funding acquisition, methodology, writing—reviewing and editing. TC: Funding acquisition, methodology, writing—reviewing and editing. EH: Funding acquisition, methodology, writing—reviewing and editing. AM: Funding acquisition, methodology, project administration, writing—reviewing and editing. ENO: Funding acquisition, methodology, writing—reviewing and editing. GE: Methodology and writing—reviewing and editing. AES: Methodology and writing—reviewing and editing. AMP: Conceptualisation, funding acquisition, investigation, methodology, project administration, writing—reviewing and editing. ASVS: Conceptualisation, formal analysis, funding acquisition, investigation, methodology, writing—original draft, writing—reviewing and editing. AE: Conceptualisation, funding acquisition, investigation, methodology, project administration, writing—reviewing and editing. PP: Conceptualisation, funding acquisition, investigation, methodology, project administration, writing—reviewing and editing.

All authors had full access to all the data in the study and had final responsibility for the decision to submit for publication. ADP and ASVS accessed and verified the data.

## Data sharing statement

Deidentified individual participant data with a data dictionary will be available on request to the corresponding author. Data access requests will be reviewed by study investigators and data sharing will require signing of a data access agreement. The study protocol and analysis code are available on the London School of Hygiene and Tropical Medicine Data Compass.

## Declaration of interests

ADP and MJ report salary support from the NIHR. AES has received speaker honoraria from Servier, Abbott, Sanofi, AstraZeneca, Medtronic, Omron, and Aktiia and serves on scientific advisory boards for Medtronic, Roche/Alnylam, AstraZeneca, Servier, SiSU Health and Sky Labs. All other authors declare no competing interests.

## Appendix A Supplementary data

Supplementary Table S22

Supplementary Figure S1 Flowchart summarising identification, screening, consent, and data availability. HDSS is Health and Demographic Surveillance System.

Supplementary Figure S2 Forest plot of target organ damage prevalence stratified by body mass index and country, across blood pressure categories. Any TOD defined as presence of at least one marker of TOD. Blood pressure categories defined using the 2024 European Society of Cardiology categories based on 24-hour ambulatory blood pressure measurement. CI is Confidence Interval.

Supplementary Figure S3 Clustering of target organ damage for all participants for cardiac and renal target organ damage markers. All potential clusters are shown in the matrices as linked dots. Prevalence of each cluster is shown in the bar graphs. Set size shows the total number of participants with that marker. Target organ damage as defined elsewhere in the paper. Blood pressure categories defined using the 2024 European Society of Cardiology categories based on 24-hour ambulatory blood pressure measurement.

IHCoR-Africa Collaborative
