# Supplementary material for: Prevalence of target organ damage in rural Kenya and The Gambia: a cross-sectional study
Source: Lancet Reg Health Afr. 2026 Sep;7:None. doi: 10.1016/j.lanafr.2026.100087 (PMC13561962; doi:10.1016/j.lanafr.2026.100087)
Supplement: Supplementary file 1 [file mmc23.pdf]

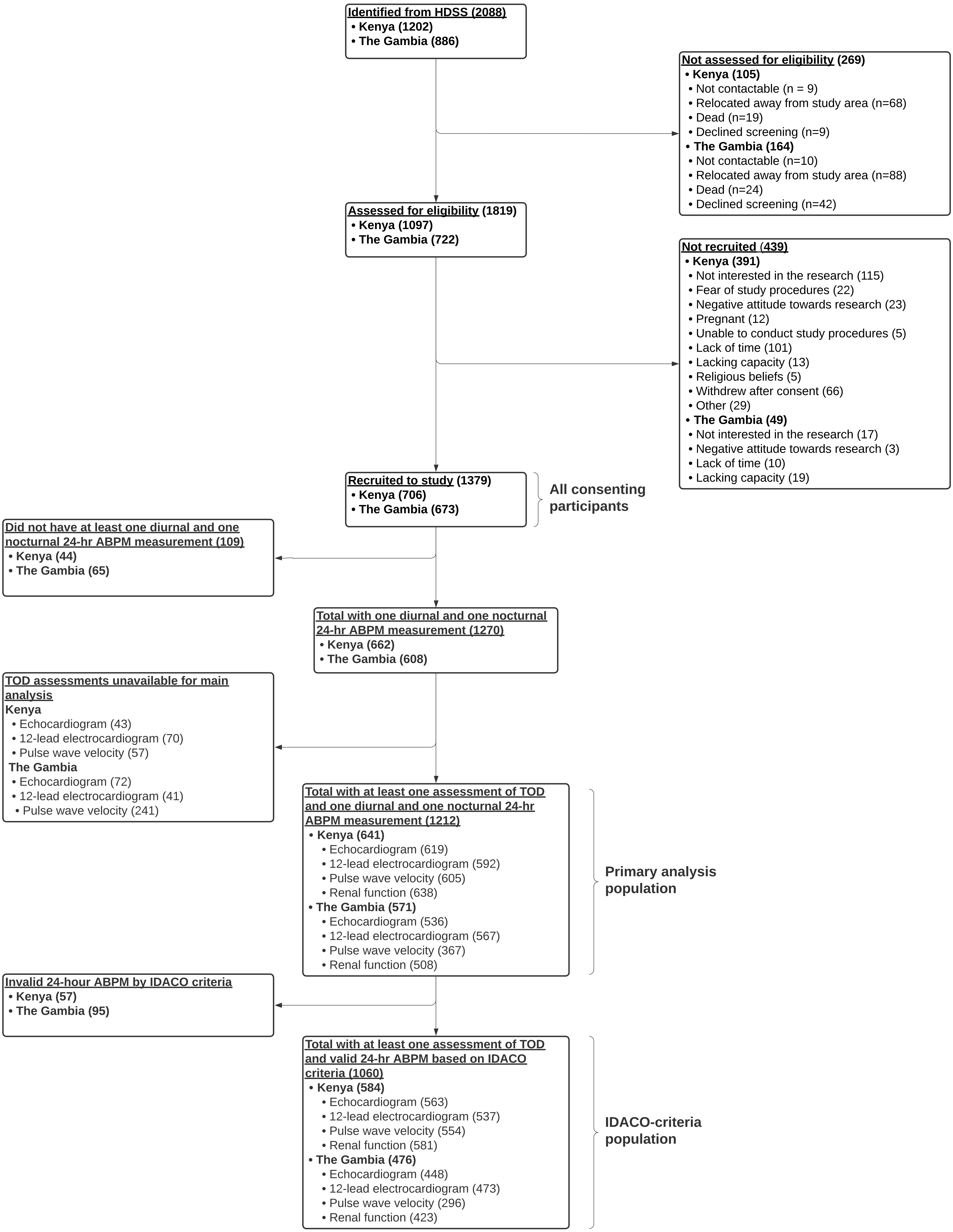
Identified from HDSS (2088)
• Kenya (1202)
• The Gambia (886)
Not assessed for eligibility (269)
• Kenya (105)
• Not contactable (n = 9)
• Relocated away from study area (n=68)
• Dead (n=19)
• Declined screening (n=9)
• The Gambia (164)
• Not contactable (n=10)
• Relocated away from study area (n=88)
• Dead (n=24)
• Declined screening (n=42)
Assessed for eligibility (1819)
• Kenya (1097)
• The Gambia (722)
Not recruited (439)
• Kenya (391)
• Not interested in the research (115)
• Fear of study procedures (22)
• Negative attitude towards research (23)
• Pregnant (12)
• Unable to conduct study procedures (5)
• Lack of time (101)
• Lacking capacity (13)
• Religious beliefs (5)
• Withdrew after consent (66)
• Other (29)
• The Gambia (49)
• Not interested in the research (17)
• Negative attitude towards research (3)
• Lack of time (10)
• Lacking capacity (19)
Recruited to study (1379)
• Kenya (706)
• The Gambia (673)
All consenting participants
Did not have at least one diurnal and one nocturnal 24-hr ABPM measurement (109)
• Kenya (44)
• The Gambia (65)
Total with one diurnal and one nocturnal 24-hr ABPM measurement (1270)
• Kenya (662)
• The Gambia (608)
TOD assessments unavailable for main analysis
Kenya
• Echocardiogram (43)
• 12-lead electrocardiogram (70)
• Pulse wave velocity (57)
The Gambia
• Echocardiogram (72)
• 12-lead electrocardiogram (41)
• Pulse wave velocity (241)
Total with at least one assessment of TOD and one diurnal and one nocturnal 24-hr ABPM measurement (1212)
• Kenya (641)
• Echocardiogram (619)
• 12-lead electrocardiogram (592)
• Pulse wave velocity (605)
• Renal function (638)
• The Gambia (571)
• Echocardiogram (536)
• 12-lead electrocardiogram (567)
• Pulse wave velocity (367)
• Renal function (508)
Primary analysis population
Invalid 24-hour ABPM by IDACO criteria
• Kenya (57)
• The Gambia (95)
Total with at least one assessment of TOD and valid 24-hr ABPM based on IDACO criteria (1060)
• Kenya (584)
• Echocardiogram (563)
• 12-lead electrocardiogram (537)
• Pulse wave velocity (554)
• Renal function (581)
• The Gambia (476)
• Echocardiogram (448)
• 12-lead electrocardiogram (473)
• Pulse wave velocity (296)
• Renal function (423)
IDACO-criteria population
