# Supplementary material for: Prevalence of target organ damage in rural Kenya and The Gambia: a cross-sectional study
Source: Lancet Reg Health Afr. 2026 Sep;7:None. doi: 10.1016/j.lanafr.2026.100087 (PMC13561962; doi:10.1016/j.lanafr.2026.100087)
Supplement: Supplementary file 2 [file mmc24.pdf]

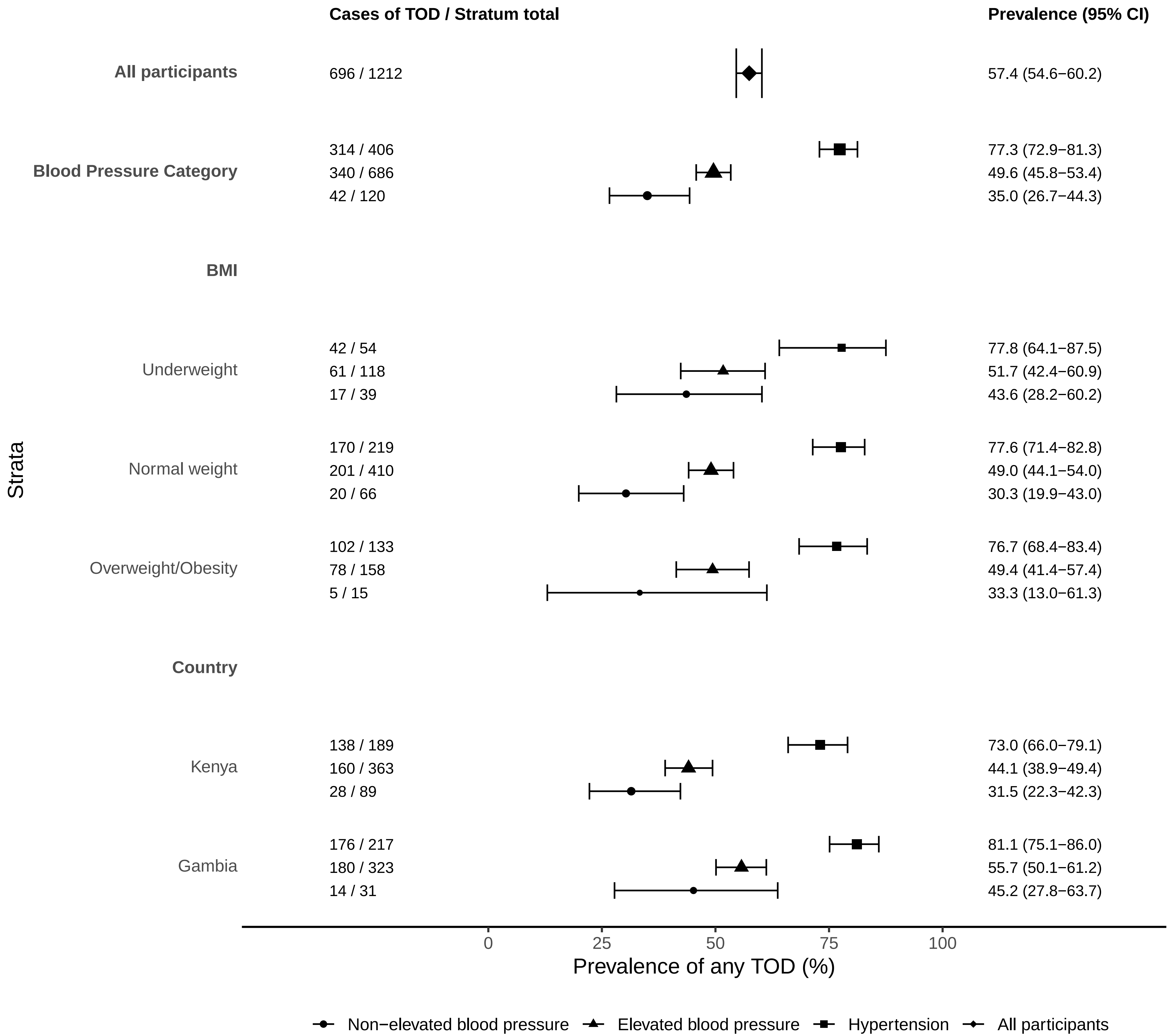

Cases of TOD / Stratum total
Prevalence (95% CI)
All participants
696 / 1212
57.4 (54.6−60.2)
Blood Pressure Category
314 / 406
77.3 (72.9−81.3)
340 / 686
49.6 (45.8−53.4)
42 / 120
35.0 (26.7−44.3)
BMI
Underweight
42 / 54
77.8 (64.1−87.5)
61 / 118
51.7 (42.4−60.9)
17 / 39
43.6 (28.2−60.2)
Normal weight
170 / 219
77.6 (71.4−82.8)
201 / 410
49.0 (44.1−54.0)
20 / 66
30.3 (19.9−43.0)
Overweight/Obesity
102 / 133
76.7 (68.4−83.4)
78 / 158
49.4 (41.4−57.4)
5 / 15
33.3 (13.0−61.3)
Country
Kenya
138 / 189
73.0 (66.0−79.1)
160 / 363
44.1 (38.9−49.4)
28 / 89
31.5 (22.3−42.3)
Gambia
176 / 217
81.1 (75.1−86.0)
180 / 323
55.7 (50.1−61.2)
14 / 31
45.2 (27.8−63.7)
Strata
0
25
50
75
100
Prevalence of any TOD (%)
Non−elevated blood pressure
Elevated blood pressure
Hypertension
All participants
