# Supplementary material for: Prevalence of target organ damage in rural Kenya and The Gambia: a cross-sectional study
Source: Lancet Reg Health Afr. 2026 Sep;7:None. doi: 10.1016/j.lanafr.2026.100087 (PMC13561962; doi:10.1016/j.lanafr.2026.100087)
Supplement: Supplementary file 4 [file mmc1.docx]

Table S1: Participant characteristics stratified by country. Categorical data reported as counts and percentages. Continuous data reported as mean and standard deviation. @ indicates self-reported data.

|  | **All Participants** | **Kenya** | **The Gambia** |
| --- | --- | --- | --- |
| **n** | 1,212 | 641 | 571 |
| **Demographics and anthropometrics** |  |  |  |
| Age in years, mean (SD)^@^ | 56·8 (14·8) | 57·2 (15·1) | 56·5 (14·5) |
| Female, n (%)^@^ | 762 (62·9) | 422 (65·8) | 340 (59·5) |
| Body mass index in kg/m², mean (SD) | 22·6 (4·4) | 22·4 (4·5) | 22·7 (4·4) |
| Formal schooling in years, mean (SD)^@^ | 4·6 (4·7) | 4·3 (4·5) | 4·8 (4·8) |
| In employment, n (%)^@^ | 1034 (86·1) | 534 (84·8) | 500 (87·6) |
| **Haemodynamics (24-hour ABPM)** |  |  |  |
| Heart rate in beats per minute, mean (SD) | 73·1 (9·4) | 71·7 (9·0) | 74·6 (9·6) |
| Systolic blood pressure in mmHg, mean (SD) | 121·0 (18·1) | 119·5 (18·1) | 122·7 (18·0) |
| Diastolic blood pressure in mmHg, mean (SD) | 74·3 (9·2) | 73·0 (9·4) | 75·6 (8·8) |
| **Blood biochemistry and lipids** |  |  |  |
| Serum potassium in mmol/L, mean (SD) | 4·1 (0·8) | 4·4 (0·6) | 3·7 (0·9) |
| Serum sodium in mmol/L, mean (SD) | 139·5 (6·2) | 138·8 (7·9) | 140·3 (3·0) |
| Serum cholesterol in mmol/L, mean (SD) | 4·5 (1·1) | 4·6 (1·1) | 4·5 (1·0) |
| Serum triglycerides in mmol/L, mean (SD) | 1·0 (0·5) | 1·1 (0·6) | 0·9 (0·5) |
| Serum LDL cholesterol in mmol/L, mean (SD) | 2·7 (0·9) | 2·8 (1·0) | 2·7 (0·9) |
| Serum creatinine in µmol/L, mean (SD) | 69·5 (30·2) | 74·2 (36·0) | 63·5 (19·2) |
| **Medical history^@^** |  |  |  |
| Tobacco use, n (%) | 140 (11·6) | 79 (12·3) | 61 (10·7) |
| Previous cardiovascular disease, n (%) | 43 (3·6) | 30 (4·7) | 13 (2·3) |
| Heart disease, n (%) | 9 (0·7) | 9 (1·4) | 0 (0·0) |
| Heart attack, n (%) | 15 (1·3) | 14 (2·3) | 1 (0·2) |
| Stroke, n (%) | 21 (1·7) | 9 (1·4) | 12 (2·1) |
| Diabetes, n (%) | 26 (2·2) | 9 (1·4) | 17 (3·0 |
| Kidney disease, n (%) | 11 (0·9) | 8 (1·3) | 3 (0·5) |
| High cholesterol, n (%) | 96 (8·0) | 4 (0·6) | 92 (16·1) |
| Asthma, n (%) | 47 (3·9) | 34 (5·3) | 13 (2·3) |
| Tuberculosis, n (%) | 18 (1·5) | 8 (1·2) | 10 (1·8) |
| Arthritis, n (%) | 29 (2·4) | 5 (0·8) | 29 (2·4) |
| HIV, n (%) | 26 (2·1) | 24 (3·8) | 2 (0·4) |
| Other chronic conditions, n (%) | 5 (1·2) | 13 (2·0) | 2 (0·4) |
