# Supplementary material for: Prevalence of target organ damage in rural Kenya and The Gambia: a cross-sectional study
Source: Lancet Reg Health Afr. 2026 Sep;7:None. doi: 10.1016/j.lanafr.2026.100087 (PMC13561962; doi:10.1016/j.lanafr.2026.100087)
Supplement: Supplementary file 5 [file mmc10.docx]

Table S10: Continuous target organ damage variables stratified by country. Results reported as mean and standard deviation. P-values are from analysis of variance between strata.

|  | **Overall** | **Kenya** | **The Gambia** |  | **p-value** |
| --- | --- | --- | --- | --- | --- |
|  | 1,212 | 641 | 571 |  | - |
| LV mass in g, (mean, SD) | 118·7 (41·6) | 126·2 (45·9) | 110·0 (34·2) |  | <0·001 |
| Indexed LV mass to allometric growth rate, g/m2·7 (mean, SD) | 33·5 (11·8) | 37·3 (12·8) | 29·1 (8·7) |  | <0·001 |
| Indexed LV mass to body surface area, g/m2 (mean, SD) | 74·2 (24·1) | 81·2 (26·2) | 66·1 (18·3) |  | <0·001 |
| Global longitudinal strain, % (mean, SD) | -18·6 (2·7) | -19·1 (2·8) | -17·9 (2·5) |  | <0·001 |
| Left ventricular ejection fraction, % (mean, SD) | 56·8 (6·3) | 59·0 (5·8) | 54·1 (5·9) |  | <0·001 |
| Pulse wave velocity, m/s (mean, SD) | 9·7 (1·6) | 9·4 (1·4) | 10·1 (1·8) |  | <0·001 |
| Estimated glomerular filtration rate, mL/min/1·73m^2^ (mean, SD) | 96·4 (17·6) | 92·2 (18·2) | 101·6 (15·4) |  | <0·001 |
