# Supplementary material for: Prevalence of target organ damage in rural Kenya and The Gambia: a cross-sectional study
Source: Lancet Reg Health Afr. 2026 Sep;7:None. doi: 10.1016/j.lanafr.2026.100087 (PMC13561962; doi:10.1016/j.lanafr.2026.100087)
Supplement: Supplementary file 6 [file mmc11.docx]

Table S11: Continuous target organ damage variables stratified by sex. Results reported as mean and standard deviation. P-values are from analysis of variance between strata.

|  | **Overall** | **Men** | **Women** |  | **p-value** |
| --- | --- | --- | --- | --- | --- |
|  | 1,212 | 450 | 762 |  | - |
| LV mass, g (mean, SD) | 118·7 (41·6) | 134·5 (45·7) | 109·3 (35·8) |  | <0·001 |
| Indexed LV mass to allometric growth rate, g/m^2·7^ (mean, SD) | 33·5 (11·8) | 34·0 (12·2) | 33·1 (11·6) |  | 0·242 |
| Indexed LV mass to body surface area, g/m^2^ (mean, SD) | 74·2 (24·1) | 80·3 (25·5) | 70·5 (22·4) |  | <0·001 |
| Global longitudinal strain, % (mean, SD) | -18·6 (2·7) | -17·9 (2·8) | -19·0 (2·6) |  | <0·001 |
| Left ventricular ejection fraction, % (mean, SD) | 56·8 (6·3) | 55·7 (7·0) | 57·4 (5·9) |  | <0·001 |
| Pulse wave velocity, m/s (mean, SD) | 9·7 (1·6) | 9·6 (1·5) | 9·7 (1·6) |  | 0·366 |
| Estimated glomerular filtration rate, mL/min/1·73m^2^ (mean, SD) | 96·4 (17·6) | 94·5 (18·3) | 97·5 (17·2) |  | 0·006 |
