# Supplementary material for: Prevalence of target organ damage in rural Kenya and The Gambia: a cross-sectional study
Source: Lancet Reg Health Afr. 2026 Sep;7:None. doi: 10.1016/j.lanafr.2026.100087 (PMC13561962; doi:10.1016/j.lanafr.2026.100087)
Supplement: Supplementary file 7 [file mmc12.docx]

Table S12: Continuous target organ damage variables stratified by age. Results reported as mean and standard deviation. P-values are from analysis of variance between strata.

|  | **Overall** | **30-39** | **40-49** | **50-59** | **60-69** | **70+** | **p-value** |
| --- | --- | --- | --- | --- | --- | --- | --- |
|  | 1,212 | 202 | 231 | 254 | 253 | 272 | - |
| LV mass, g (mean, SD) | 118·7 (41·6) | 106·7 (31·1) | 117·6 (41·4) | 118·7 (42·1) | 122·4 (41·6) | 125·4 (46·5) | <0·001 |
| Indexed LV mass to allometric growth rate, g/m^2·7^ (mean, SD) | 33·5 (11·8) | 29·2 (8·5) | 32·1 (11·8) | 32·7 (11·1) | 34·8 (11·5) | 37·4 (13·5) | <0·001 |
| Indexed LV mass to body surface area, g/m^2^ (mean, SD) | 74·2 (24·1) | 65·7 (17·7) | 71·2 (24·5) | 72·0 (21·6) | 77·2 (23·3) | 82·3 (27·7) | <0·001 |
| Global longitudinal strain, % (mean, SD) | -18·6 (2·7) | -19·3 (2·3) | -19·3 (2·3) | -19·1 (2·6) | -18·0 (2·5) | -17·2 (3·2) | <0·001 |
| Left ventricular ejection fraction, % (mean, SD) | 56·8 (6·3) | 56·7 (5·2) | 57·8 (4·9) | 58·0 (5·8) | 56·5 (6·3) | 55·1 (8·1) | <0·001 |
| Pulse wave velocity, m/s (mean, SD) | 9·7 (1·6) | 8·6 (1·4) | 9·3 (1·6) | 9·7 (1·3) | 10·0 (1·5) | 10·1 (1·7) | <0·001 |
| Estimated glomerular filtration rate, mL/min/1·73m^2^ (mean, SD) | 96·4 (17·6) | 110·7 (15·7) | 105·6 (12·6) | 97·9 (13·2) | 90·7 (15·4) | 81·8 (14·6) | <0·001 |
