# Supplementary material for: Prevalence of target organ damage in rural Kenya and The Gambia: a cross-sectional study
Source: Lancet Reg Health Afr. 2026 Sep;7:None. doi: 10.1016/j.lanafr.2026.100087 (PMC13561962; doi:10.1016/j.lanafr.2026.100087)
Supplement: Supplementary file 8 [file mmc13.docx]

Table S13: Continuous target organ damage variables stratified by systolic blood pressure in 20mmHg groups. Results reported as mean and standard deviation. P-values are from analysis of variance between strata. Systolic blood pressure based on 24-hour ambulatory blood pressure measurement.

|  | **Overall** | **80-99mmHg** | **100-119 mmHg** | **120-139 mmHg** | **140-159 mmHg** | **160-179 mmHg** | **180-199 mmHg** | **>=200 mmHg** | **p-value** |  |
| --- | --- | --- | --- | --- | --- | --- | --- | --- | --- | --- |
|  | 1,212 | 77 | 592 | 366 | 129 | 34 | 11 | 3 | - |  |
| LV mass, g (mean, SD) | 118·7 (41·6) | 93·4 (24·6) | 109·6 (38·6) | 125·3 (36·9) | 143·5 (48·4) | 150·0 (45·7) | 152·7 (38·1) | 279·4 (70·0) | <0·001 |  |
| Indexed LV mass to allometric growth rate, g/m^2·7^ (mean, SD) | 33·5 (11·8) | 27·6 (8·6) | 30·9 (10·8) | 35·0 (10·8) | 40·8 (13·8) | 42·4 (13·3) | 42·9 (10·1) | 69·7 (26·8) | <0·001 |  |
| Indexed LV mass to body surface area, g/m^2^ (mean, SD) | 74·2 (24·1) | 65·1 (17·1) | 68·9 (22·9) | 77·0 (21·5) | 88·3 (25·8) | 92·5 (28·9) | 95·3 (20·3) | 153·2 (56·6) | <0·001 |  |
| Global longitudinal strain, % (mean, SD) | -18·6 (2·7) | -19·9 (2·5) | -19·3 (2·4) | -18·0 (2·7) | -16·5 (2·5) | -16·3 (2·8) | -15·2 (1·2) | -12·6 (0·3) | <0·001 |  |
| Left ventricular ejection fraction, % (mean, SD) | 56·8 (6·3) | 58·9 (5·2) | 57·5 (5·8) | 56·5 (6·7) | 53·9 (6·7) | 54·2 (7·4) | 52·0 (5·0) | 42·4 (2·8) | <0·001 |  |
| Pulse wave velocity, m/s (mean, SD) | 9·7 (1·6) | 9·1 (1·4) | 9·4 (1·6) | 9·9 (1·5) | 10·1 (1·7) | 10·0 (1·7) | 11·7 (2·0) | 9·8 (2·7) | <0·001 |  |
| Estimated glomerular filtration rate, mL/min/1·73m^2^ (mean, SD) | 96·4 (17·6) | 99·3 (17·2) | 100·6 (16·0) | 93·8 (17·4) | 86·2 (19·7) | 88·2 (13·9) | 84·3 (11·7) | 77·7 (34·6) | <0·001 |  |
