# Supplementary material for: Prevalence of target organ damage in rural Kenya and The Gambia: a cross-sectional study
Source: Lancet Reg Health Afr. 2026 Sep;7:None. doi: 10.1016/j.lanafr.2026.100087 (PMC13561962; doi:10.1016/j.lanafr.2026.100087)
Supplement: Supplementary file 9 [file mmc14.docx]

Table S14: Left ventricular hypertrophy assessment using alternative definitions. For LVH-BSA and LVH-IVSd, p-values is Chi-square test of independence. For continuous variables, p-values are from analysis of variance between strata. BSA is body surface area.

|  | **Overall** | **Kenya** | | **The Gambia** | **p-value** |
| --- | --- | --- | --- | --- | --- |
|  | 1,212 | 641 | | 571 | - |
| LVH-BSA, n (%) | 120 (10·4) | 105 (17·0) | | 15 ( 2·8) | <0·001 |
| LVH-IVSd, n (%) | 147 (12·7) | 78 (12·6) | | 69 (12·9) | 0·960 |
| LV mass in g, mean (SD) | 118·7 (41·6) | 126·2 (45·9) | | 110·0 (34·2) | <0·001 |
| Height in cm, mean (SD) | 160·2 (9·1) | 157·1 (8·3) | | 163·7 (8·6) | <0·001 |
| Weight in kg, mean (SD) | 58·0 (12·7) | 55·4 (12·6) | | 60·8 (12·1) | <0·001 |
| Body surface area in m^2^, mean (SD) | 1·6 (0·2) | 1·5 (0·2) | | 1·7 (0·2) | <0·001 |
| Indexed LV mass to body surface area in g/m^2^, mean (SD) | 74·2 (24·1) | 81·2 (26·2) | 66·1 (18·3) |  | <0·001 |
| Interventricular septal thickness at end-diastole in cm, mean (SD) | 0·8 (0·2) | 0·9 (0·2) | 0·8 (0·2) |  | <0·001 |
