# Supplementary material for: Prevalence of target organ damage in rural Kenya and The Gambia: a cross-sectional study
Source: Lancet Reg Health Afr. 2026 Sep;7:None. doi: 10.1016/j.lanafr.2026.100087 (PMC13561962; doi:10.1016/j.lanafr.2026.100087)
Supplement: Supplementary file 10 [file mmc15.docx]

Table S15: Participant characteristics for all consented participants, the primary analysis population, and the IDACO population. Categorical data reported as counts and percentages. Continuous data reported as mean and standard deviation. @ indicates self-reported data.

|  | **All consented participants** | **Primary analysis population** | **IDACO-criteria population** |
| --- | --- | --- | --- |
|  | 1,379 | 1,212 | 1,060 |
| **Demographics and anthropometrics** |  |  |  |
| Age in years, mean (SD) ^@^ | 57·0 (15·0) | 56·8 (14·8) | 56·6 (14·8) |
| Female, n (%)^@^ | 858 (62·4) | 762 (62·9) | 686 (64·7) |
| Country | - | - | - |
| Kenya | 706 (51·2) | 641 (52·9) | 584 (55·1) |
| The Gambia | 673 (48·8) | 571 (47·1) | 476 (44·9) |
| BMI in kg/m², mean (SD) | 22·6 (4·5) | 22·6 (4·4) | 22·6 (4·5) |
| Formal schooling in years, mean (SD)^@^ | 4·5 (4·6) | 4·6 (4·7) | 4·6 (4·6) |
| In employment, n (%)^@^ | 1111 (86·3) | 1034 (86·1) | 896 (85·4) |
| **Haemodynamics (24-hour ABPM)** |  |  |  |
| Heart rate in bpm, mean (SD) | 72·1 (13·5) | 73·1 (9·4) | 73·1 (9·3) |
| Systolic blood pressure in mmHg, mean (SD) | 121·3 (18·5) | 121·0 (18·1) | 121·0 (17·9) |
| Diastolic blood pressure in mmHg, mean (SD) | 73·2 (13·6) | 74·3 (9·2) | 74·2 (9·0) |
| **Blood biochemistry and lipids** |  |  |  |
| Serum potassium in mmol/L, mean (SD) | 4·1 (0·8) | 4·1 (0·8) | 4·1 (0·8) |
| Serum sodium in mmol/L, mean (SD) | 139·5 (6·2) | 139·5 (6·2) | 139·4 (6·4) |
| Serum cholesterol in mmol/L, mean (SD) | 4·6 (1·1) | 4·5 (1·1) | 4·5 (1·1) |
| Serum triglycerides in mmol/L, mean (SD) | 1·0 (0·5) | 1·0 (0·5) | 1·0 (0·6) |
| Serum LDL cholesterol in mmol/L, mean (SD) | 2·7 (0·9) | 2·7 (0·9) | 2·8 (0·9) |
| Serum creatinine in µmol/L, mean (SD) | 69·4 (29·6) | 69·5 (30·2) | 69·0 (28·8) |
| **Medical history^@^** |  |  |  |
| Tobacco use, n (%) | 152 (11·7) | 140 (11·6) | 116 (11·0) |
| Previous cardiovascular disease, n (%) | 45 (3·5) | 43 (3·6) | 40 (3·8) |
| Heart disease, n (%) | 10 (0·8) | 9 (0·7) | 9 (0·8) |
| Heart attack, n (%) | 15 (1·2) | 15 (1·3) | 14 (1·4) |
| Stroke, n (%) | 22 (1·7) | 21 (1·7) | 19 (1·8) |
| Diabetes, n (%) | 27 (2·1) | 26 (2·2) | 23 (2·2) |
| Kidney disease, n (%) | 12 (0·9) | 11 (0·9) | 10 (0·9) |
| High cholesterol, n (%) | 107 (8·3) | 96 (8·0) | 79 (7·5) |
| Asthma, n (%) | 49 (3·8) | 47 (3·9) | 45 (4·2) |
| Tuberculosis, n (%) | 20 (1·5) | 18 (1·5) | 17 (1·6) |
| Arthritis, n (%) | 33 (2·5) | 29 (2·4) | 26 (2·5) |
| HIV, n (%) | 29 (2·2) | 26 (2·1) | 23 (2·2) |
| Other chronic conditions, n (%) | 17 (1·3) | 15 (1·2) | 13 (1·2) |
