# Supplementary material for: Prevalence of target organ damage in rural Kenya and The Gambia: a cross-sectional study
Source: Lancet Reg Health Afr. 2026 Sep;7:None. doi: 10.1016/j.lanafr.2026.100087 (PMC13561962; doi:10.1016/j.lanafr.2026.100087)
Supplement: Supplementary file 11 [file mmc16.docx]

Table S16: Sensitivity analysis of target organ damage prevalence stratified by blood pressure category using the IDACO-criteria population. For all prevalence estimates, the denominator is participants with an available measurement for the relevant TOD marker, excluding missing data. P-values are Chi-square test of independence, or Fisher’s Exact test if expected frequency was less than 5, between strata. Blood pressure categories defined using the 2024 European Society of Cardiology categories based on 24-hour ambulatory blood pressure measurement.

|  | **Overall** | **Non-elevated blood pressure** | **Elevated blood pressure** | **Hypertension** | **p-value** |  |
| --- | --- | --- | --- | --- | --- | --- |
|  | 1,060 | 100 | 603 | 357 | - |  |
| **LVH-ECG, n (%)** |  |  |  |  |  |  |
| Absent | 703 (69·6) | 84 (86·6) | 432 (75·1) | 187 (55·3) | - |  |
| All LVH-ECG | 307 (30·4) | 13 (13·4) | 143 (24·9) | 151 (44·7) | <0·001 |  |
| LVH-ECG without strain | 257 (25·4) | 11 (11·3) | 125 (21·7) | 121 (35·8) | - |  |
| LVH-ECG with strain | 50 (5·0) | 2 (2·1) | 18 (3·1) | 30 (8·9) | - |  |
| Missing | 50 | 3 | 28 | 19 | - |  |
| **LVH-Echo, n (%)** | 103 (10·2) | 5 (5·4) | 34 (5·9) | 64 (18·7) | <0·001 |  |
| Missing | 49 | 7 | 27 | 15 | - |  |
| **Global Longitudinal Strain, n (%)** |  |  |  |  |  |  |
| Normal | 588 (60·1) | 78 (83·9) | 375 (67·7) | 135 (40·7) | - |  |
| Borderline | 230 (23·5) | 11 (11·8) | 125 (22·6) | 94 (28·3) | - |  |
| Reduced | 161 (16·4) | 4 (4·3) | 54 (9·7) | 103 (31·0) | <0·001 |  |
| Missing | 81 | 7 | 49 | 25 | - |  |
| **LV Systolic Dysfunction, n (%)** |  |  |  |  |  |  |
| Normal | 880 (90·8) | 93 (100·0) | 518 (93·8) | 269 (83·0) | - |  |
| Mildly Reduced or Reduced | 89 (9·2) | 0 (0·0) | 34 (6·2) | 55 (17·0 | <0·001 |  |
| Missing | 91 | 7 | 51 | 33 | - |  |
| **Arterial Stiffness (n, %)** | 321 (37·8) | 19 (21·6) | 154 (33·0) | 148 (50·0) | <0·001 |  |
| Missing | 210 | 12 | 137 | 61 | - |  |
| **Renal impairment (n, %)** | 38 (3·8) | 2 (2·1) | 12 (2·1) | 24 (7·3) | <0·001 |  |
| Missing | 56 | 3 | 27 | 26 | - |  |
| **Any TOD, n (%)** | 604 (57·0) | 33 (33·0) | 297 (49·3) | 274 (76·8) | <0·001 |  |
