# Supplementary material for: Prevalence of target organ damage in rural Kenya and The Gambia: a cross-sectional study
Source: Lancet Reg Health Afr. 2026 Sep;7:None. doi: 10.1016/j.lanafr.2026.100087 (PMC13561962; doi:10.1016/j.lanafr.2026.100087)
Supplement: Supplementary file 12 [file mmc17.docx]

Table S17: Sensitivity analysis of continuous target organ damage variables stratified by blood pressure category using the IDACO-criteria population. Results reported as mean and standard deviation. P-values are from analysis of variance between strata. Blood pressure categories defined using the 2024 European Society of Cardiology categories based on 24-hour ambulatory blood pressure measurement.

|  | **Overall** | **Non-elevated blood pressure** | **Elevated blood pressure** | **Hypertension** | **p-value** |  |
| --- | --- | --- | --- | --- | --- | --- |
|  | 1,060 | 100 | 603 | 357 | - |  |
| LV mass, g (mean, SD) | 118·8 (41·5) | 108·4 (35·8) | 111·2 (37·5) | 134·5 (44·9) | <0·001 |  |
| Indexed LV mass to allometric growth rate, g/m^2·7^ (mean, SD) | 33·6 (11·9) | 31·5 (9·7) | 31·3 (10·9) | 37·9 (12·9) | <0·001 |  |
| Indexed LV mass to body surface area, g/m^2^ (mean, SD) | 74·2 (24·0) | 71·6 (20·9) | 69·7 (22·0) | 82·5 (25·9) | <0·001 |  |
| Global longitudinal strain, % (mean, SD) | -18·6 (2·7) | -20·3 (2·2) | -19·2 (2·4) | -17·2 (2·7) | <0·001 |  |
| Left ventricular ejection fraction, % (mean, SD) | 56·9 (6·2) | 59·7 (4·4) | 57·6 (5·8) | 55·0 (6·6) | <0·001 |  |
| Pulse wave velocity, m/s (mean, SD) | 9·6 (1·6) | 8·9 (1·7) | 9·5 (1·5) | 10·1 (1·7) | <0·001 |  |
| Estimated glomerular filtration rate, mL/min/1·73m2(mean, SD) | 96·4 (17·5) | 97·9 (16·3) | 99·1 (15·8) | 91·3 (19·3) | <0·001 |  |
