# Supplementary material for: Prevalence of target organ damage in rural Kenya and The Gambia: a cross-sectional study
Source: Lancet Reg Health Afr. 2026 Sep;7:None. doi: 10.1016/j.lanafr.2026.100087 (PMC13561962; doi:10.1016/j.lanafr.2026.100087)
Supplement: Supplementary file 13 [file mmc18.docx]

Table S18: Age- and sex – standardised prevalence of target organ damage stratified by blood pressure category. For all prevalence estimates, the denominator is participants with an available measurement for the relevant target organ damage marker, excluding missing data. P-values are Chi-square test of independence, or Fisher’s Exact test if expected frequency was less than 5, between the stratified groups. Blood pressure categories defined using the 2024 European Society of Cardiology categories based on 24-hour ambulatory blood pressure measurement. Standardised to the WHO 2000-2025 World Standard Population.

|  | **Overall** | **Non-elevated blood pressure** | **Elevated blood pressure** | **Hypertension** |  |
| --- | --- | --- | --- | --- | --- |
| **LVH-ECG, %** |  |  |  |  |  |
| Absent | 72·8 | 90·3 | 76·8 | 56·9 |  |
| All LVH-ECG | 27·2 | 9·7 | 23·2 | 43·1 |  |
| LVH-ECG without strain | 23·2 | 8·2 | 20·4 | 35·2 |  |
| LVH-ECG with strain | 4·0 | 1·5 | 2·7 | 7·9 |  |
| **LVH-Echo, %** | 7·1 | 4·2 | 4·7 | 13·7 |  |
| **Global Longitudinal Strain, %** |  |  |  |  |  |
| Normal | 64·9 | 83·8 | 70·7 | 44·6 |  |
| Borderline | 22·9 | 13·8 | 21·0 | 30·7 |  |
| Reduced | 12·2 | 2·4 | 8·3 | 24·7 |  |
| **LV Systolic Dysfunction, %** |  |  |  |  |  |
| Normal | 92·6 | 99·7 | 94·0 | 86·7 |  |
| Mildly Reduced or Reduced | 7·4 | 0·3 | 6·0 | 13·3 |  |
| **Arterial Stiffness, %** | 32·0 | 18·5 | 28·6 | 44·9 |  |
| **Renal impairment, %** | 2·3 | 1·1 | 1·7 | 4·2 |  |
| **Any TOD, %** | 50·0 | 26·8 | 44·3 | 71·3 |  |
