# Supplementary material for: Prevalence of target organ damage in rural Kenya and The Gambia: a cross-sectional study
Source: Lancet Reg Health Afr. 2026 Sep;7:None. doi: 10.1016/j.lanafr.2026.100087 (PMC13561962; doi:10.1016/j.lanafr.2026.100087)
Supplement: Supplementary file 14 [file mmc19.docx]

Table S19: Age- and sex – standardised continuous target organ damage variables stratified by blood pressure category. Results reported as mean and standard deviation. P-values are from analysis of variance between strata. Blood pressure categories defined using the 2024 European Society of Cardiology categories based on 24-hour ambulatory blood pressure measurement. Standardised to the WHO 2000-2025 World Standard Population.

|  | **Overall** | **Non-elevated blood pressure** | **Elevated blood pressure** | **Hypertension** |  |
| --- | --- | --- | --- | --- | --- |
|  | 1,212 | 120 | 686 | 406 |  |
| LV mass, g (mean, SD) | 116·1 (39·6) | 106·9 (32·1) | 110·4 (38) | 132·2 (41·3) |  |
| Indexed LV mass to allometric growth rate, g/m^2·7^ (mean, SD) | 32·2 (11·1) | 31 (9) | 30·6 (10·7) | 36·2 (11·6) |  |
| Indexed LV mass to body surface area, g/m^2^ (mean, SD) | 71·5 (22·5) | 70·1 (18·7) | 68·4 (22·1) | 78·9 (23·1 |  |
| Global longitudinal strain, % (mean, SD) | -18·9 (2·5) | -20·3 (2·2) | -19·3 (2·3) | -17·6 (2·6) |  |
| Left ventricular ejection fraction, % (mean, SD) | 57·1 (5·8) | 59·2 (4·4) | 57·5 (5·6) | 55·6 (6·1) |  |
| Pulse wave velocity, m/s (mean, SD) | 9·4 (1·6) | 8·7 (1·5) | 9·3 (1·5) | 10 (1·6) |  |
| Estimated glomerular filtration rate, mL/min/1·73m2 (mean, SD) | 96·4 (17·5) | 97·9 (16·3) | 99·1 (15·8) | 91·3 (19·3) |  |
