# Supplementary material for: Prevalence of target organ damage in rural Kenya and The Gambia: a cross-sectional study
Source: Lancet Reg Health Afr. 2026 Sep;7:None. doi: 10.1016/j.lanafr.2026.100087 (PMC13561962; doi:10.1016/j.lanafr.2026.100087)
Supplement: Supplementary file 15 [file mmc2.docx]

Table S2: Participant characteristics stratified by country. Blood pressure categories defined using the 2024 European Society of Cardiology categories based on 24-hour ambulatory blood pressure measurement. Categorical data reported as counts and percentages. Continuous data reported as mean and standard deviation. @ indicates self-reported data.

|  | **All Participants** | **Male** | **Female** |
| --- | --- | --- | --- |
| **n** | 1,212 | 450 | 762 |
| **Demographics and anthropometrics** |  |  |  |
| Age in years, mean (SD)^@^ | 56·8 (14·8) | 57·7 (14·6) | 56·3 (14·9) |
| BMI in kg/m², mean (SD) | 22·6 (4·4) | 21·7 (3·8) | 23·0 (4·7) |
| Formal schooling in years, mean (SD)^@^ | 4·6 (4·7) | 6·6 (5·0) | 3·4 (4·0) |
| In employment, n (%)^@^ | 1034 (86·1) | 410 (91·9) | 624 (82·6) |
| **Haemodynamics (24-hour ABPM)** |  |  |  |
| Heart rate in bpm, mean (SD) | 73·1 (9·4) | 70·2 (9·1) | 74·8 (9·1) |
| Systolic blood pressure in mmHg, mean (SD) | 121·0 (18·1) | 123·8 (18·2) | 119·4 (17·9) |
| Diastolic blood pressure in mmHg, mean (SD) | 74·3 (9·2) | 75·9 (9·4) | 73·3 (9·0) |
| **Blood biochemistry and lipids** |  |  |  |
| Serum potassium in mmol/L, mean (SD) | 4·1 (0·8) | 4·1 (0·9) | 4·1 (0·7) |
| Serum sodium in mmol/L, mean (SD) | 139·5 (6·2) | 139·1 (4·9) | 139·7 (6·9) |
| Serum cholesterol in mmol/L, mean (SD) | 4·5 (1·1) | 4·3 (1·0) | 4·7 (1·1) |
| Serum triglycerides in mmol/L, mean (SD) | 1·0 (0·5) | 1·0 (0·5) | 1·0 (0·6) |
| Serum LDL cholesterol in mmol/L, mean (SD) | 2·7 (0·9) | 2·5 (0·9) | 2·9 (0·9) |
| Serum creatinine in µmol/L, mean (SD) | 69·5 (30·2) | 82·8 (37·8) | 61·5 (21·0) |
| **Medical history^@^** |  |  |  |
| Tobacco use, n (%) | 140 (11·6) | 78 (17·4) | 62 (8·1) |
| Previous cardiovascular disease, n (%) | 43 (3·6) | 20 (4·5) | 23 (3·0) |
| Heart disease, n (%) | 9 (0·7) | 4 (0·9) | 5 (0·7) |
| Heart attack, n (%) | 15 (1·3) | 4 (0·9) | 11 (1·5) |
| Stroke, n (%) | 21 (1·7) | 13 (2·9) | 8 (1·1) |
| Diabetes, n (%) | 26 (2·2) | 11 (2·4) | 15 (2·0) |
| Kidney disease, n (%) | 11 (0·9) | 5 (1·1) | 6 (0·8) |
| High cholesterol, n (%) | 96 (8·0) | 32 (7·1) | 64 (8·5) |
| Asthma, n (%) | 47 (3·9) | 13 (2·9) | 34 (4·5) |
| Tuberculosis, n (%) | 18 (1·5) | 9 (2·0) | 9 (1·2) |
| Arthritis, n (%) | 29 (2·4) | 13 (2·9) | 16 (2·1) |
| HIV, n (%) | 26 (2·1) | 10 (2·2) | 16 (2·1) |
| Other chronic conditions, n (%) | 15 (1·2) | 5 (1·1) | 10 (1·3) |
