# Supplementary material for: Prevalence of target organ damage in rural Kenya and The Gambia: a cross-sectional study
Source: Lancet Reg Health Afr. 2026 Sep;7:None. doi: 10.1016/j.lanafr.2026.100087 (PMC13561962; doi:10.1016/j.lanafr.2026.100087)
Supplement: Supplementary file 16 [file mmc20.docx]

Table S20: Age- and sex – standardised prevalence of target organ damage stratified by country for the primary analysis population. For all prevalence estimates, the denominator is participants with an available measurement for the relevant target organ damage marker, excluding missing data. P-values are Chi-square test of independence, or Fisher’s Exact test if expected frequency was less than 5, between the stratified groups. Standardised to the WHO 2000-2025 World Standard Population.

|  | **Overall** | **Kenya** | **The Gambia** |  |
| --- | --- | --- | --- | --- |
| **LVH-ECG, n (%)** |  |  |  |  |
| Absent | 72·8 | 82·7 | 62·6 |  |
| All LVH-ECG | 27·2 | 17·3 | 37·4 |  |
| LVH-ECG without strain | 23·2 | 15·4 | 31·1 |  |
| LVH-ECG with strain | 4 | 1·8 | 6·3 |  |
| **LVH-Echo, n (%)** | 7·1 | 12·1 | 1·5 |  |
| **Global Longitudinal Strain, n (%)** |  |  |  |  |
| Normal | 64·9 | 71·2 | 57·6 |  |
| Borderline | 22·9 | 20·3 | 25·9 |  |
| Reduced | 12·2 | 8·5 | 16·5 |  |
| **LV Systolic Dysfunction, n (%)** |  |  |  |  |
| Normal | 92·6 | 96·9 | 87·7 |  |
| Mildly Reduced or Reduced | 7·4 | 3·1 | 12·3 |  |
| **Arterial Stiffness (n, %)** | 32 | 26·2 | 42·8 |  |
| **Renal impairment (n, %)** | 2·3 | 3·0 | 1·4 |  |
| **Any TOD, n (%)** | 50·0 | 44.2 | 56.3 |  |
