# Supplementary material for: Prevalence of target organ damage in rural Kenya and The Gambia: a cross-sectional study
Source: Lancet Reg Health Afr. 2026 Sep;7:None. doi: 10.1016/j.lanafr.2026.100087 (PMC13561962; doi:10.1016/j.lanafr.2026.100087)
Supplement: Supplementary file 17 [file mmc21.docx]

Table S21: Age- and sex – standardised continuous target organ damage variables stratified by country. Results reported as mean and standard deviation. P-values are from analysis of variance between strata. Standardised to the WHO 2000-2025 World Standard Population.

|  | **Overall** | **Kenya** | **The Gambia** |  |
| --- | --- | --- | --- | --- |
|  | 1,212 | 641 | 571 |  |
| LV mass, g (mean, SD) | 116·1 (39·6) | 123·4 (44·7) | 107·8 (31) |  |
| Indexed LV mass to allometric growth rate, g/m^2·7^ (mean, SD) | 32·2 (11·1) | 35·8 (12·3) | 28·1 (7·7) |  |
| Indexed LV mass to body surface area, g/m^2^ (mean, SD) | 71·5 (22·5) | 78·2 (25·1) | 64 (16·3) |  |
| Global longitudinal strain, % (mean, SD) | -18·9 (2·5) | -19·5 (2·5) | -18·3 (2·4) |  |
| Left ventricular ejection fraction, % (mean, SD) | 57·1 (5·8) | 59·2 (5·3) | 54·8 (5·4) |  |
| Pulse wave velocity, m/s (mean, SD) | 9·4 (1·6) | 9·2 (1·4) | 9·8 (1·7) |  |
| Estimated glomerular filtration rate, mL/min/1·73m2 (mean, SD) | 101·3 (16·6) | 97·1 (17·1) | 106·3 (14·3) |  |
