# Supplementary material for: Prevalence of target organ damage in rural Kenya and The Gambia: a cross-sectional study
Source: Lancet Reg Health Afr. 2026 Sep;7:None. doi: 10.1016/j.lanafr.2026.100087 (PMC13561962; doi:10.1016/j.lanafr.2026.100087)
Supplement: Supplementary file 18 [file mmc22.docx]

Prevalence of target organ damage clusters stratified by country for cardiac and renal target organ damage markers. P-values are Chi-square test of independence, or Fisher’s Exact test if expected frequency was less than 5, between the stratified groups.

|  | **Overall** | **Kenya** | **The Gambia** | **p-value** |
| --- | --- | --- | --- | --- |
| All participants, n (%) | 938 | 521 | 417 | - |
| No markers of TOD, n (%) | 513 (54·7) | 324 (62·2) | 189 (45·3) | <0·001 |
| One marker of TOD, n (%) | 270 (28·8) | 127 (24·4) | 143 (34·3) | 0·001 |
| Two markers of TOD, n (%) | 94 (10·0) | 42 ( 8·1) | 52 (12·5) | 0·034 |
| Three markers of TOD, n (%) | 47 ( 5·0) | 20 ( 3·8) | 27 ( 6·5) | 0·091 |
| Four markers of TOD, n (%) | 13 ( 1·4) | 7 ( 1·3) | 6 ( 1·4) | 1·000 |
| Five markers of TOD, n (%) | 1 ( 0·1) | 1 ( 0·2) | 0 ( 0·0) | 1·000 |
