# Supplementary material for: Prevalence of target organ damage in rural Kenya and The Gambia: a cross-sectional study
Source: Lancet Reg Health Afr. 2026 Sep;7:None. doi: 10.1016/j.lanafr.2026.100087 (PMC13561962; doi:10.1016/j.lanafr.2026.100087)
Supplement: Supplementary file 19 [file mmc26.docx]

# The IHCoR-Africa Collaborative

| **Name** | **Affiliation** |
| --- | --- |
| Benjamin Tsofa | KEMRI-Wellcome Trust Research Programme, Kenya |
| Edwine Barasa | KEMRI-Wellcome Trust Research Programme, Kenya |
| Robinson Oyando | KEMRI-Wellcome Trust Research Programme, Kenya |
| Samson Kinyanjui | KEMRI-Wellcome Trust Research Programme, Kenya |
| Noni Mumba | KEMRI-Wellcome Trust Research Programme, Kenya |
| Jemima Kamano | Moi University, Kenya |
| Violet Naanyu | Moi University, Kenya |
| Lilian Mbau | Kenya Cardiac Society, Kenya |
| Nadia Aliyan | Ministry of Health and Medical Services, Kenya |
| Nancy Kagwanja | KEMRI-Wellcome Trust Research Programme, Kenya |
| James Abuje | KEMRI-Wellcome Trust Research Programme, Kenya |
| Assan Jaye | Medical Research Council Unit The Gambia at LSHTM, The Gambia |
| Lamin Jobarteh | Medical Research Council Unit The Gambia at LSHTM, The Gambia |
| Saidina Ceesay | Medical Research Council Unit The Gambia at LSHTM, The Gambia |
| Ellen Nolte | London School of Hygiene & Tropical Medicine, United Kingdom |
| Melanie Morris | London School of Hygiene & Tropical Medicine, United Kingdom |
| David Prieto-Merino | London School of Hygiene & Tropical Medicine, United Kingdom |
| Cova Bascaran | London School of Hygiene & Tropical Medicine, United Kingdom |
| Syreen Hassan | London School of Hygiene & Tropical Medicine, United Kingdom |
| Mavis Foster-Nyarko | London School of Hygiene & Tropical Medicine, United Kingdom |
