# Supplementary material for: Prevalence of target organ damage in rural Kenya and The Gambia: a cross-sectional study
Source: Lancet Reg Health Afr. 2026 Sep;7:None. doi: 10.1016/j.lanafr.2026.100087 (PMC13561962; doi:10.1016/j.lanafr.2026.100087)
Supplement: Supplementary file 20 [file mmc27.docx]

Prevalence of cardiovascular target organ damage in rural Kenya and the Gambia: a cross-sectional study

**Supplementary Information**

Authors:

Alexander D Perkins, Modou Jobe, Ruth Lucinde, Juliet Otieno Awori, Sampa Sanneh, Catherine Kalu, Clement Mwagwabi, Elminah Saru, Gabriela Miani, Kieran Mwazo, David A Leon, Tim Clayton, Emily Herrett, Adrianna Murphy, Elijah Nyainda Ogola, Georg Ehret, Aletta E Schutte, Andrew M Prentice, Anoop SV Shah, Anthony Etyang, Pablo Perel for The IHCoR-Africa Collaborative

Table of Contents

[Sample size calculations	2](#_Toc227593633)

[Blood pressure measurement and definitions	2](#_Toc227593634)

[Target organ damage measurement	3](#_Toc227593635)

[Cross-country data collection standardisation	4](#_Toc227593636)

[Target organ damage definitions	4](#_Toc227593637)

[Left ventricular hypertrophy	4](#_Toc227593638)

[Global longitudinal strain	5](#_Toc227593639)

[Left ventricular systolic dysfunction	5](#_Toc227593640)

[Arterial stiffness	5](#_Toc227593641)

[Renal dysfunction	5](#_Toc227593642)

[Accuracy of LV-Mass Indexing	6](#_Toc227593643)

[Referral of participants with hypertension	6](#_Toc227593644)

[Supplementary Figures	6](#_Toc227593645)

[Supplementary Tables	7](#_Toc227593646)

[Supplementary References	10](#_Toc227593647)

# Sample size calculations

The sample size of 1250 participants was calculated for the accurate estimation of hypertension prevalence in the study population as part of the wider aims of the IHCoR-Africa project. Assuming a prevalence of hypertension of 25–35%, as detected by the reference standard method (24-hr-ABPM), we estimated that we would require 832–1003 participants to generate validity parameters for hypertension prevalence with a precision of ±3% overall and ±5% at each of the two sites. We have made a provision for loss of data in 25% of participants due to poor data quality, taking the total number of participants required to 1250, equally divided between Kilifi (N=625) and Kiang West (N=625).

We conservatively assumed a combined prevalence of TOD of 35%. Based on the available sample size of 1250 participants, and assuming a confidence level of 95%, this allowed us to estimate TOD prevalence with an overall precision of ±2.6% and ±3.7% in each country.

# Blood pressure measurement and definitions

We conducted 24-hour automated blood pressure measurement (24-hr ABPM) using Spacelabs OnTrak devices (Spacelabs, Snoqualmie, USA). Measurements were conducted by trained community health workers and community health nurses following a standard operating procedure to ensure comparability across study sites. Participants had an appropriately sized cuff placed on their non-dominant arm, and received instructions on how to care for the device. Devices were programmed to record a measurement every 15 minutes during the day (0600 and 2200) and every 30 minutes at night (2200-0600). Following completion of the 24-hour ABPM, study staff collected the devices and downloaded the data for verification.

To be included in the primary analysis, participants had to have at least one successful diurnal measurement (between 0600 to 2200) and one successful nocturnal measurement (between 2200 to 0600).

For the sensitivity analysis using the International Database of Ambulatory Blood Pressure in relation to Cardiovascular Outcome (IDACO) criteria, participants had to have at least 10 successful diurnal measurements, 5 successful nocturnal measurements, and overall success rate of 70%.^1^

For both analyses, participants were categorised using mean 24-hour systolic and diastolic blood pressure as defined by the 2024 European Society of Cardiology guidelines.^2^ These categories were Non-elevated blood pressure (SBP < 115 mmHg and DBP < 65mmHg), Elevated blood pressure (115mmHg ≤ SBP < 130mmHg and DBP < 80mmHg, or 65mmHg ≤ DBP < 80mmHg and SBP < 130mmHg), and Hypertension (SBP ≥ 130mmHg or DBP ≥ 80mmHg).

# Target organ damage measurement

Target organ damage (TOD) was assessed following 24-hour ABPM. All TOD measurements were conducted on the same day for each participant. To ensure comparability, all data acquisition was guided by aligned standard operating procedures, and staff were extensively trained on all study procedures.

#### Electrocardiography

A standard 12-lead resting electrocardiogram (ECG) was acquired using a MAC-5 device in the Gambia (GE Healthcare, Chicago, Illinois, USA) and a Edan 601-E device in Kenya (Edan Instruments Inc, Shenzen, China). ECGs were conducted by experienced cardiologists and cardiac technicians. Participants were supine for the assessment in a climate controlled room.

#### Echocardiography

Comprehensive echocardiography studies were conducted using the Vivid iq Ultrasound System in both countries (GE Healthcare, Chicago, Illinois, USA) by trained and experienced cardiologists and cardiac technicians. Echocardiography studies included assessment of cardiac chamber dimensions and volumes, cardiac function and global longitudinal strain (GLS). Interpretation and evaluation of echocardiograms was standardised and included automated measurements were applicable. Intra-ventricular septal thickness (IVSd) was measured at end-diastole using standardised approaches. GLS was determined by automated measurements of peak systolic GLS by speckle-tracking on 2-dimensional echocardiographic scans. Left ventricular mass was calculated using the Devereux formula.^3^

#### Pulse wave velocity

We measured aortic pulse wave velocity (PWV) using the Arteriograph 24 (TensioMed, Budapest, Hungary).^4^ PWV was determined based on the distance from the jugular to the symphysis and the time difference between the direct systolic wave passing the brachial blood pressure cuff and the reflected systolic wave returning in the opposite direction.

#### Estimated glomerular filtration rate

Blood samples were collected and serum creatinine was determined using the fixed-time Jaffe reaction in both sites at Good Clinical Laboratory Practice accredited facilities. Estimated glomerular filtration rate was calculated from serum creatinine using the 2021 CKD-EPI formula without adjustment for race.^5,6^

# Cross-country data collection standardisation

To ensure comparability between the two research sites, data collection procedures were standardised. Standard operating procedures were developed with input from both sites, and training was harmonised. Where possible, automated measurements were taken to reduce measurement bias. Furthermore, alignment of data collection procedures was bolstered by direct exchange between the teams in Kenya and The Gambia including reciprocal field visits.

# Target organ damage definitions

### Left ventricular hypertrophy

Collected ECGs were independently reported and manually coded by cardiologists. LVH-ECG which was defined by meeting one of the following criteria: Sokolow-Lyon (SVI+RV5 > 35 mm), Cornell (SV3+RaVL > 28 mm for men, and SV3+RaVL > 20 mm for women) or Romholt-Estes criteria.^7–9^ Minnesota coding showed repolarization abnormalities was used for classification of LVH-ECG without strain (Minnesota codes 3.1 and 3.3) and LVH-ECG with strain (Minnesota Codes 3.1 or 3.3 plus 4.1, 4.2, 4.3 or 4.4).^10^ All forms of LVH-ECG were classified as TOD.

The primary definition of LVH-Echo was based on left ventricular mass (LVM) indexed to the allometric growth rate of height to the power of 2.7 with sex-specific thresholds of >50g/m^2.7^ in men, and >47g/m^2.7^ in women.^2^ Echocardiographic LVH was also categorised based on LVM indexed to body surface area (LVH-BSA) with sex specific thresholds of 115g/m^2^ in men, and 95g/m^2^ in women.^2,11^ Finally, a crude measure of LVH based on interventricular septal thickness (LVH-IVSd) was included using sex-specific thresholds. For men, mild LVH-IVSd was an IVSd of 1.1–1.3 cm, moderate LVH-IVSd as 1.4–1.6 cm, and severe LVH-IVSd was ≥ 1.7 cm. For women, mild LVH-IVSd was an IVSd of 1.0–1.2 cm, moderate LVH-IVSd was 1.3–1.5 cm, and severe LVH-IVSd was ≥ 1.6 cm.^11^ For LVH-IVSd, moderate and severe was considered to be TOD.

### Global longitudinal strain

GLS was classified as Normal if less than -18%, Borderline if between -18 and -16%, and Reduced of greater than -16%. Only reduced GLS was classified as TOD.^12^

### Left ventricular systolic dysfunction

LVSD was assessed by Simpson’s biplane left ventricular ejection fraction (LVEF): reduced (LVEF ≤40%), mildly reduced (LVEF 41-49%), normal (LVEF≥50%).^13^ LVSD was classified as TOD if LVEF was mildly reduced or reduced.

### Arterial stiffness

Arterial stiffness was determined by PWV using the clinical guideline threshold of 10 m/s. PWV ≥10 m/s was considered arterial stiffness.^2^

### Renal dysfunction

Renal dysfunction was determined based on eGFR using the recommended hypertension clinical guideline threshold of 60mL/min/1.73m^2^.^2^

# Accuracy of LV-Mass Indexing

Guidelines for hypertension manangement recommend using LV mass indexed (LVMI) to either the allometric growth rate (height^2.7^) or body surface area (BSA) to assess LVH by echocardiography (LVH-Echo and LVH-BSA in our study). However, in our study, LVH-Echo and LVH-BSA prevalence in The Gambia was significantly lower compared to other assessments of LVH (LVH-ECG and LVH-IVSd) in The Gambia. Furthermore prevalence of LVH-Echo and LVH-BSA were much lower than the prevalence of LVH-Echo or LVH-BSA in Kenya, while for LVH-ECG and LVH-IVSd, prevalence was higher in the Gambia than Kenya (Tables S4 and S11). These results suggests that HIC-derived thresholds for CV-TOD, and guideline recommend assessments methods, may not be appropriate in some sSA contexts.

# Referral of participants with hypertension

Participants identified as having hypertension during the study were referred to the local community health nurse or to their local clinic for further treatment. It was not possible to systematically track the precise number who took up the referral as we were not able to monitor attendance at the clinic over an extended period. However, based on our study team’s integration with the local healthcare system, anecdotal evidence suggests that the majority of those referred sought care.

# Supplementary Figures

**Figure S1:** Flowchart summarising identification, screening, consent, and data availability. HDSS is Health and Demographic Surveillance System.

**Figure S2:** Forest plot of target organ damage prevalence stratified by body mass index and country, across blood pressure categories. Any TOD defined as presence of at least one marker of TOD. Blood pressure categories defined using the 2024 European Society of Cardiology categories based on 24-hour ambulatory blood pressure measurement. CI is Confidence Interval.

**Figure S3:** Clustering of target organ damage for all participants for cardiac and renal target organ damage markers. All potential clusters are shown in the matrices as linked dots. Prevalence of each cluster is shown in the bar graphs. Set size shows the total number of participants with that marker. Target organ damage as defined elsewhere in the paper. Blood pressure categories defined using the 2024 European Society of Cardiology categories based on 24-hour ambulatory blood pressure measurement.

# Supplementary Tables

**Table S1:** Participant characteristics stratified by country. Categorical data reported as counts and percentages. Continuous data reported as mean and standard deviation. @ indicates self-reported data.

**Table S2:** Participant characteristics stratified by country. Categorical data reported as counts and percentages. Continuous data reported as mean and standard deviation. @ indicates self-reported data.

**Table S3:** Participant characteristics stratified by age. Categorical data reported as counts and percentages. Continuous data reported as mean and standard deviation. @ indicates self-reported data.

**Table S4:** Participant characteristics stratified by systolic blood pressure in 20mmHg groups. Blood pressure categories defined using the 2024 European Society of Cardiology categories based on 24-hour ambulatory blood pressure measurement. Categorical data reported as counts and percentages. Continuous data reported as mean and standard deviation. @ indicates self-reported data.

**Table S5:** Prevalence of target organ damage stratified by country for the primary analysis population. For all prevalence estimates, the denominator is participants with an available measurement for the relevant target organ damage marker, excluding missing data. P-values are Chi-square test of independence, or Fisher’s Exact test if expected frequency was less than 5, between the stratified groups.

**Table S6:** Prevalence of target organ damage stratified by sex for the primary analysis population. For all prevalence estimates, the denominator is participants with an available measurement for the relevant target organ damage marker, excluding missing data. P-values are Chi-square test of independence, or Fisher’s Exact test if expected frequency was less than 5, between the stratified groups.

**Table S7:** Prevalence of target organ damage stratified by age for the primary analysis population. For all prevalence estimates, the denominator is participants with an available measurement for the relevant target organ damage marker, excluding missing data. P-values are Chi-square test of independence, or Fisher’s Exact test if expected frequency was less than 5, between the stratified groups.

**Table S8:** Prevalence of target organ damage stratified by systolic blood pressure in 20mmHg groups for the primary analysis population. For all prevalence estimates, the denominator is participants with an available measurement for the relevant target organ damage marker, excluding missing data. P-values are Chi-square test of independence, or Fisher’s Exact test if expected frequency was less than 5, between the stratified groups. Systolic blood pressure based on 24-hour ambulatory blood pressure measurement.

**Table S9:** Continuous target organ damage variables stratified by blood pressure category. Results reported as mean and standard deviation. P-values are from analysis of variance between strata. Blood pressure categories defined using the 2024 European Society of Cardiology categories based on 24-hour ambulatory blood pressure measurement.

**Table S10:** Continuous target organ damage variables stratified by country. Results reported as mean and standard deviation. P-values are from analysis of variance between strata.

**Table S11:** Continuous target organ damage variables stratified by sex. Results reported as mean and standard deviation. P-values are from analysis of variance between strata.

**Table S12:** Continuous target organ damage variables stratified by age. Results reported as mean and standard deviation. P-values are from analysis of variance between strata.

**Table S13:** Continuous target organ damage variables stratified by systolic blood pressure in 20mmHg groups. Results reported as mean and standard deviation. P-values are from analysis of variance between strata. Systolic blood pressure based on 24-hour ambulatory blood pressure measurement.

**Table S14**: Left ventricular hypertrophy assessment using alternative definitions. For LVH-BSA and LVH-IVSd, p-values is Chi-square test of independence. For continuous variables, p-values are from analysis of variance between strata. BSA is body surface area.

**Table S15:** Participant characteristics for all consented participants, the primary analysis population, and the IDACO population. Categorical data reported as counts and percentages. Continuous data reported as mean and standard deviation. @ indicates self-reported data.

**Table S16:** Sensitivity analysis of target organ damage prevalence stratified by blood pressure category using the IDACO-criteria population. For all prevalence estimates, the denominator is participants with an available measurement for the relevant TOD marker, excluding missing data. P-values are Chi-square test of independence, or Fisher’s Exact test if expected frequency was less than 5, between strata. Blood pressure categories defined using the 2024 European Society of Cardiology categories based on 24-hour ambulatory blood pressure measurement.

**Table S17:** Sensitivity analysis of continuous target organ damage variables stratified by blood pressure category using the IDACO-criteria population. Results reported as mean and standard deviation. P-values are from analysis of variance between strata. Blood pressure categories defined using the 2024 European Society of Cardiology categories based on 24-hour ambulatory blood pressure measurement.

**Table S18:** Age- and sex – standardised prevalence of target organ damage stratified by blood pressure category. For all prevalence estimates, the denominator is participants with an available measurement for the relevant target organ damage marker, excluding missing data. P-values are Chi-square test of independence, or Fisher’s Exact test if expected frequency was less than 5, between the stratified groups. Blood pressure categories defined using the 2024 European Society of Cardiology categories based on 24-hour ambulatory blood pressure measurement. Standardised to the WHO 2000-2025 World Standard Population.

**Table S19:** Age- and sex – standardised continuous target organ damage variables stratified by blood pressure category. Results reported as mean and standard deviation. P-values are from analysis of variance between strata. Blood pressure categories defined using the 2024 European Society of Cardiology categories based on 24-hour ambulatory blood pressure measurement. Standardised to the WHO 2000-2025 World Standard Population.

**Table S20:** Age- and sex – standardised prevalence of target organ damage stratified by country for the primary analysis population. For all prevalence estimates, the denominator is participants with an available measurement for the relevant target organ damage marker, excluding missing data. P-values are Chi-square test of independence, or Fisher’s Exact test if expected frequency was less than 5, between the stratified groups. Standardised to the WHO 2000-2025 World Standard Population.

**Table S21:** Age- and sex – standardised continuous target organ damage variables stratified by country. Results reported as mean and standard deviation. P-values are from analysis of variance between strata. Standardised to the WHO 2000-2025 World Standard Population.

**Table S22:** Prevalence of target organ damage clusters stratified by country for cardiac and renal target organ damage markers. P-values are Chi-square test of independence, or Fisher’s Exact test if expected frequency was less than 5, between the stratified groups.

# Supplementary References

1	Thijs L, Hansen TW, Kikuya M, *et al.* The International Database of Ambulatory Blood Pressure in relation to Cardiovascular Outcome (IDACO): protocol and research perspectives. *Blood Press Monit* 2007; **12**: 255–62.

2	McEvoy JW, McCarthy CP, Bruno RM, *et al.* 2024 ESC Guidelines for the management of elevated blood pressure and hypertension. *Eur Heart J* 2024; **45**: 3912–4018.

3	Devereux RB, Alonso DR, Lutas EM, *et al.* Echocardiographic assessment of left ventricular hypertrophy: comparison to necropsy findings. *Am J Cardiol* 1986; **57**: 450–8.

4	Milan A, Zocaro G, Leone D, *et al.* Current assessment of pulse wave velocity: comprehensive review of validation studies. *J Hypertens* 2019; **37**: 1547–57.

5	Fabian J, Kalyesubula R, Mkandawire J, *et al.* Measurement of kidney function in Malawi, South Africa, and Uganda: a multicentre cohort study. *Lancet Glob Health* 2022; **10**: e1159–69.

6	Inker LA, Eneanya ND, Coresh J, *et al.* New creatinine- and cystatin C-based equations to estimate GFR without race. *N Engl J Med* 2021; **385**: 1737–49.

7	Sokolow M, Lyon TP. The ventricular complex in left ventricular hypertrophy as obtained by unipolar precordial and limb leads. *Am Heart J* 1949; **37**: 161–86.

8	Casale PN, Devereux RB, Alonso DR, Campo E, Kligfield P. Improved sex-specific criteria of left ventricular hypertrophy for clinical and computer interpretation of electrocardiograms: validation with autopsy findings. *Circulation* 1987; **75**: 565–72.

9	Romhilt DW, Estes EH Jr. A point-score system for the ECG diagnosis of left ventricular hypertrophy. *Am Heart J* 1968; **75**: 752–8.

10	Blackburn H. Electrocardiographic classification for population comparisons. The Minnesota code. *J Electrocardiol* 1969; **2**: 5–9.

11	Lang RM, Bierig M, Devereux RB, *et al.* Recommendations for chamber quantification: a report from the American Society of Echocardiography’s Guidelines and Standards Committee and the Chamber Quantification Writing Group, developed in conjunction with the European Association of Echocardiography, a branch of the European Society of Cardiology. *J Am Soc Echocardiogr* 2005; **18**: 1440–63.

12	Yang H, Wright L, Negishi T, Negishi K, Liu J, Marwick TH. Research to practice: Assessment of left ventricular global longitudinal strain for surveillance of cancer chemotherapeutic-related cardiac dysfunction. *JACC Cardiovasc Imaging* 2018; **11**: 1196–201.

13	McDonagh TA, Metra M, Adamo M, *et al.* 2021 ESC Guidelines for the diagnosis and treatment of acute and chronic heart failure. *Eur Heart J* 2021; **42**: 3599–726.
