# Supplementary material for: Prevalence of target organ damage in rural Kenya and The Gambia: a cross-sectional study
Source: Lancet Reg Health Afr. 2026 Sep;7:None. doi: 10.1016/j.lanafr.2026.100087 (PMC13561962; doi:10.1016/j.lanafr.2026.100087)
Supplement: Supplementary file 21 [file mmc3.docx]

Table S3: Participant characteristics stratified by age. Categorical data reported as counts and percentages. Continuous data reported as mean and standard deviation. @ indicates self-reported data.

|  | **All Participants** | **30-39** | **40-49** | **50-59** | **60-69** | **70+** |
| --- | --- | --- | --- | --- | --- | --- |
|  | 1,212 | 202 | 231 | 254 | 253 | 272 |
| **Demographics and anthropometrics** |  |  |  |  |  |  |
| Age in years, mean (SD)^@^ | 56·8 (14·8) | 35·9 (2·3) | 45·1 (2·9) | 54·5 (2·8) | 64·7 (2·7) | 77·3 (5·2) |
| Female, n (%)^@^ | 762 (62·9) | 141 (69·8) | 146 (63·2) | 152 (59·8) | 159 (62·8) | 164 (60·3) |
| BMI in kg/m², mean (SD) | 22·6 (4·4) | 22·5 (4·0) | 23·3 (4·6) | 23·1 (4·6) | 22·4 (4·7) | 21·6 (4·1) |
| Formal schooling in years, mean (SD)^@^ | 4·6 (4·7) | 6·6 (4·2) | 5·9 (4·3) | 5·3 (4·6) | 3·5 (4·5) | 2·2 (4·2) |
| In employment, n (%)^@^ | 1034 (86·1) | 183 (90·6) | 217 (93·9) | 237 (93·3) | 214 (86·3) | 183 (68·8) |
| **Haemodynamics (24-hour ABPM)** |  |  |  |  |  |  |
| Heart rate in bpm, mean (SD) | 73·1 (9·4) | 76·2 (9·2) | 74·5 (9·2) | 73·0 (8·8) | 71·6 (9·2) | 71·0 (9·6) |
| Systolic blood pressure in mmHg, mean (SD) | 121·0 (18·1) | 113·2 (11·5) | 114·1 (13·3) | 118·0 (15·8) | 125·5 (19·4) | 131·4 (20·4) |
| Diastolic blood pressure in mmHg, mean (SD) | 74·3 (9·2) | 72·2 (7·9) | 73·6 (8·6) | 75·3 (9·2) | 75·4 (9·8) | 74·3 (10·0) |
| **Blood biochemistry and lipids** |  |  |  |  |  |  |
| Serum potassium in mmol/L, mean (SD) | 4·1 (0·8) | 4·1 (1·0) | 4·0 (0·9) | 4·1 (0·9) | 4·1 (0·6) | 4·1 (0·7) |
| Serum sodium in mmol/L, mean (SD) | 139·5 (6·2) | 139·3 (6·7) | 138·9 (6·0) | 139·8 (6·9) | 140·1 (6·1) | 139·4 (5·5) |
| Serum cholesterol in mmol/L, mean (SD) | 4·5 (1·1) | 4·0 (0·8) | 4·3 (1·0) | 4·7 (1·1) | 4·8 (1·0) | 4·9 (1·1) |
| Serum triglycerides in mmol/L, mean (SD) | 1·0 (0·5) | 0·8 (0·4) | 0·9 (0·6) | 1·1 (0·6) | 1·1 (0·5) | 1·1 (0·5) |
| Serum LDL cholesterol in mmol/L, mean (SD) | 2·7 (0·9) | 2·3 (0·7) | 2·6 (0·8) | 2·8 (1·0) | 2·9 (0·9) | 3·0 (1·0) |
| Serum creatinine in µmol/L, mean (SD) | 69·5 (30·2) | 66·1 (21·3) | 65·8 (16·0) | 69·1 (26·8) | 71·0 (36·7) | 74·1 (39·7) |
| **Medical history^@^** |  |  |  |  |  |  |
| Tobacco use, n (%) | 140 (11·6) | 12 (5·9) | 21 (9·1) | 31 (12·2) | 31 (12·3) | 45 (16·5) |
| Previous cardiovascular disease, n (%) | 43 (3·6) | 3 (1·5) | 5 (2·2) | 10 (3·9) | 11 (4·3) | 14 (5·1) |
| Heart disease, n (%) | 9 (0·7) | 1 (0·5) | 1 (0·4) | 3 (1·2) | 2 (0·8) | 2 (0·7) |
| Heart attack, n (%) | 15 (1·3) | 2 (1·0) | 2 (0·9) | 3 (1·2) | 3 (1·2) | 5 (1·9) |
| Stroke, n (%) | 21 (1·7) | 0 (0·0) | 2 (0·9) | 5 (2·0) | 6 (2·4) | 8 (3·0) |
| Diabetes, n (%) | 26 (2·2) | 1 (0·5) | 4 (1·7) | 2 (0·8) | 4 (1·6) | 15 (5·5) |
| Kidney disease, n (%) | 11 (0·9) | 1 (0·5) | 3 (1·3) | 3 (1·2) | 4 (1·6) | 0 (0·0) |
| High cholesterol, n (%) | 96 (8·0) | 10 (5·0) | 16 (7·0) | 9 (3·6) | 29 (11·5) | 32 (11·8) |
| Asthma, n (%) | 47 (3·9) | 12 (5·9) | 8 (3·5) | 13 (5·1) | 7 (2·8) | 7 (2·6) |
| Tuberculosis, n (%) | 18 (1·5) | 3 (1·5) | 3 (1·3) | 3 (1·2) | 3 (1·2) | 6 (2·2) |
| Arthritis, n (%) | 29 (2·4) | 1 (0·5) | 5 (2·2) | 8 (3·1) | 7 (2·8) | 8 (2·9) |
| HIV, n (%) | 26 (2·1) | 3 (1·5) | 5 (2·2) | 10 (3·9) | 7 (2·8) | 1 (0·4) |
| Other chronic conditions, n (%) | 15 (1·2) | 3 (1·5) | 1 (0·4) | 5 (2·0) | 3 (1·2) | 3 (1·1) |
