# Supplementary material for: Prevalence of target organ damage in rural Kenya and The Gambia: a cross-sectional study
Source: Lancet Reg Health Afr. 2026 Sep;7:None. doi: 10.1016/j.lanafr.2026.100087 (PMC13561962; doi:10.1016/j.lanafr.2026.100087)
Supplement: Supplementary file 22 [file mmc4.docx]

Table S4: Participant characteristics stratified by systolic blood pressure in 20mmHg groups. Blood pressure categories defined using the 2024 European Society of Cardiology categories based on 24-hour ambulatory blood pressure measurement. Categorical data reported as counts and percentages. Continuous data reported as mean and standard deviation. @ indicates self-reported data.

|  | Overall | 80-99mmHg | 100-119 mmHg | 120-139 mmHg | 140-159 mmHg | 160-179 mmHg | 180-199 mmHg | >=200 mmHg |
| --- | --- | --- | --- | --- | --- | --- | --- | --- |
|  | 1,212 | 77 | 592 | 366 | 129 | 34 | 11 | 3 |
| **Demographics and anthropometrics** |  |  |  |  |  |  |  |  |
| Age in years, mean (SD) | 57·3 (14·8) | 53·3 (13·3) | 52·6 (13·6) | 60·7 (14·7) | 67·4 (12·2) | 68·8 (10·9) | 71·0 (9·7) | 70·8 (8·2) |
| Female, n (%) | 762 (62·9) | 60 (77·9) | 392 (66·2) | 215 (58·7) | 69 (53·5) | 20 (58·8) | 5 (45·5) | 1 (33·3) |
| Country | - | - | - | - | - | - | - | - |
| Kenya | 641 (52·9) | 57 (74·0) | 314 (53·0) | 185 (50·5) | 62 (48·1) | 17 (50·0) | 5 (45·5) | 1 (33·3) |
| The Gambia | 571 (47·1) | 20 (26·0) | 278 (47·0) | 181 (49·5) | 67 (51·9) | 17 (50·0) | 6 (54·5) | 2 (66·7) |
| Body mass index in kg/m², mean (SD) | 22·6 (4·4) | 19·0 (2·9) | 22·3 (4·0) | 23·2 (4·7) | 23·4 (5·1) | 23·3 (5·1) | 22·7 (4·8) | 24·5 (3·1) |
| Formal schooling in years^@^, mean (SD)^@^ | 4·6 (4·7) | 4·6 (4·2) | 5·0 (4·7) | 4·3 (4·7) | 3·9 (4·7) | 3·0 (5·2) | 2·3 (3·4) | 4·7 (5·0) |
| In employment, n (%)^@^ | 1034 (86·1) | 65 (84·4) | 536 (90·8) | 300 (82·6) | 99 (78·6) | 23 (74·2) | 9 (81·8) | 2 (66·7) |
| **Haemodynamics (24-hour ABPM)** |  |  |  |  |  |  |  |  |
| Heart rate in bpm, mean (SD) | 73·1 (9·4) | 72·1 (9·8) | 73·6 (9·3) | 73·1 (9·0) | 72·6 (9·0) | 69·9 (12·1) | 70·1 (14·9) | 66·0 (6·1) |
| Systolic blood pressure in mmHg, mean (SD) | 121·0 (18·1) | 95·2 (3·3) | 110·2 (5·1) | 127·7 (5·5) | 147·9 (5·6) | 166·8 (5·8) | 183·8 (3·1) | 204·0 (4·6) |
| Diastolic blood pressure in mmHg, mean (SD) | 74·3 (9·2) | 63·2 (4·0) | 70·2 (5·4) | 77·1 (6·8) | 84·3 (7·9) | 90·3 (12·4) | 99·0 (12·9) | 104·0 (11·3) |
| **Blood biochemistry and lipids** |  |  |  |  |  |  |  |  |
| Serum potassium in mmol/L, mean (SD) | 4·1 (0·8) | 4·2 (0·6) | 4·1 (0·8) | 4·1 (0·8) | 4·0 (0·7) | 3·7 (0·8) | 3·9 (0·6) | 4·1 (0·3) |
| Serum sodium in mmol/L, mean (SD) | 139·5 (6·2) | 137·6 (7·1) | 139·5 (6·2) | 139·8 (6·1) | 140·0 (6·2) | 139·9 (5·7) | 136·3 (7·1) | 139·3 (5·1) |
| Serum cholesterol in mmol/L, mean (SD) | 4·5 (1·1) | 4·3 (1·0) | 4·5 (1·0) | 4·7 (1·1) | 4·6 (1·1) | 5·0 (1·5) | 4·5 (0·7) | 5·4 (0·8) |
| Serum triglycerides in mmol/L, mean (SD) | 1·0 (0·5) | 0·9 (0·4) | 0·9 (0·4) | 1·1 (0·6) | 1·1 (0·8) | 1·2 (0·5) | 1·0 (0·5) | 0·8 (0·1) |
| Serum LDL cholesterol in mmol/L, mean (SD) | 2·7 (0·9) | 2·5 (0·9) | 2·7 (0·9) | 2·8 (1·0) | 2·8 (0·8) | 3·1 (1·3) | 2·7 (0·5) | 3·9 (0·8) |
| Serum creatinine in µmol/L, mean (SD) | 69·5 (30·2) | 65·3 (17·6) | 67·2 (27·0) | 70·3 (26·4) | 78·8 (53·2) | 70·2 (16·3) | 75·7 (16·6) | 95·0 (56·0) |
| **Medical history^@^** |  |  |  |  |  |  |  |  |
| Tobacco use, n (%) | 140 (11·6) | 8 (10·4) | 64 (10·8) | 45 (12·3) | 14 (10·9) | 8 (23·5) | 1 (9·1) | 0 (0·0) |
| Previous cardiovascular disease, n (%) | 43 (3·6) | 2 (2·6) | 16 (2·7) | 15 (4·1) | 8 (6·2) | 1 (2·9) | 1 (9·1) | 0 (0·0) |
| Heart disease, n (%) | 9 (0·7) | 1 (1·3) | 4 (0·7) | 4 (1·1) | 0 (0·0) | 0 (0·0) | 0 (0·0) | 0 (0·0) |
| Heart attack, n (%) | 15 (1·3) | 1 (1·4) | 7 (1·2) | 3 (0·8) | 4 (3·3) | 0 (0·0) | 0 (0·0) | 0 (0·0) |
| Stroke, n (%) | 21 (1·7) | 0 (0·0) | 6 (1·0) | 9 (2·5) | 4 (3·1) | 1 (3·0) | 1 (9·1) | 0 (0·0) |
| Diabetes, n (%) | 26 (2·2) | 0 (0·0) | 6 (1·0) | 11 (3·0) | 3 (2·3) | 3 (8·8) | 2 (18·2) | 1 (33·3) |
| Kidney disease, n (%) | 11 (0·9) | 1 (1·3) | 5 (0·8) | 4 (1·1) | 1 (0·8) | 0 (0·0) | 0 (0·0) | 0 (0·0) |
| High cholesterol, n (%) | 96 (8·0) | 0 (0·0) | 21 (3·6) | 41 (11·2) | 20 (15·6) | 9 (26·5) | 3 (27·3) | 2 (66·7) |
| Asthma, n (%) | 47 (3·9) | 6 (7·8) | 23 (3·9) | 12 (3·3) | 6 (4·7) | 0 (0·0) | 0 (0·0) | 0 (0·0) |
| Tuberculosis, n (%) | 18 (1·5) | 1 (1·3) | 8 (1·4) | 6 (1·6) | 3 (2·3) | 0 (0·0) | 0 (0·0) | 0 (0·0) |
| Arthritis, n (%) | 29 (2·4) | 1 (1·3) | 13 (2·2) | 10 (2·7) | 5 (3·9) | 0 (0·0) | 0 (0·0) | 0 (0·0) |
| HIV, n (%) | 26 (2·1) | 3 (3·9) | 16 (2·7) | 6 (1·6) | 1 (0·8) | 0 (0·0) | 0 (0·0) | 0 (0·0) |
| Other chronic conditions, n (%)· | 15 (1·2) | 1 (1·3) | 8 (1·4) | 4 (1·1) | 1 (0·8) | 0 (0·0) | 1 (9·1) | 0 (0·0) |
