# Supplementary material for: Prevalence of target organ damage in rural Kenya and The Gambia: a cross-sectional study
Source: Lancet Reg Health Afr. 2026 Sep;7:None. doi: 10.1016/j.lanafr.2026.100087 (PMC13561962; doi:10.1016/j.lanafr.2026.100087)
Supplement: Supplementary file 24 [file mmc6.docx]

Table S6: Prevalence of target organ damage stratified by sex for the primary analysis population. For all prevalence estimates, the denominator is participants with an available measurement for the relevant target organ damage marker, excluding missing data. P-values are Chi-square test of independence, or Fisher’s Exact test if expected frequency was less than 5, between the stratified groups.

|  | **Overall** | **Men** | **Women** | **p-value** |  |
| --- | --- | --- | --- | --- | --- |
|  | 1,212 | 450 | 762 | - |  |
| **LVH-ECG, n (%)** |  |  |  |  |  |
| Absent | 799 (68·9) | 271 (63·6) | 528 (72·0) | - |  |
| All LVH-ECG | 360 (31·1) | 155 (36·4) | 205 (28·0) | 0·004 |  |
| LVH-ECG without strain | 297 (25·6) | 123 (28·9) | 174 (23·7) | - |  |
| LVH-ECG with strain | 63 (5·4) | 32 (7·5) | 31 (4·2) | - |  |
| Missing | 53 | 24 | 29 | - |  |
| **LVH-Echo, n (%)** | 115 (10·0) | 42 (9·7) | 73 (10·1) | 0·917 |  |
| Missing | 57 | 18 | 39 | - |  |
| **Global Longitudinal Strain, n (%)** |  |  |  |  |  |
| Normal | 662 (59·6) | 199 (48·3) | 463 (66·2) | - |  |
| Borderline | 263 (23·7) | 115 (27·9) | 148 (21·2) | - |  |
| Reduced | 186 (16·7) | 98 (23·8) | 88 (12·6) | <0·001 |  |
| Missing | 101 | 38 | 63 | - |  |
| **LV Systolic Dysfunction, n (%)** |  |  |  |  |  |
| Normal | 999 (90·2) | 352 (85·9) | 647 (92·7) | - |  |
| Mildly Reduced or Reduced | 109 (9·8) | 58 (14·1) | 51 (7·3) | <0·001 |  |
| Missing | 104 | 40 | 64 | - |  |
| **Arterial Stiffness (n, %)** | 369 (38·0) | 129 (36·4) | 240 (38·8) | 0·502 |  |
| Missing | 240 | 96 | 144 | - |  |
| **Renal impairment (n, %)** | 44 (3·8) | 23 (5·4) | 21 (2·9) | 0·055 |  |
| Missing | 66 | 96 | 144 | - |  |
| **Any TOD, n (%)** | 696 (57·4) | 284 (63·1) | 412 (54·1) | 0·003 |  |
