# Supplementary material for: Prevalence of target organ damage in rural Kenya and The Gambia: a cross-sectional study
Source: Lancet Reg Health Afr. 2026 Sep;7:None. doi: 10.1016/j.lanafr.2026.100087 (PMC13561962; doi:10.1016/j.lanafr.2026.100087)
Supplement: Supplementary file 26 [file mmc8.docx]

Table S8: Prevalence of target organ damage stratified by systolic blood pressure in 20mmHg groups for the primary analysis population. For all prevalence estimates, the denominator is participants with an available measurement for the relevant target organ damage marker, excluding missing data. P-values are Chi-square test of independence, or Fisher’s Exact test if expected frequency was less than 5, between the stratified groups. Systolic blood pressure based on 24-hour ambulatory blood pressure measurement.

|  | **Overall** | **80-99mmHg** | **100-119 mmHg** | **120-139 mmHg** | **140-159 mmHg** | **160-179 mmHg** | **180-199 mmHg** | **>=200 mmHg** | **p-value** |  |
| --- | --- | --- | --- | --- | --- | --- | --- | --- | --- | --- |
|  | 1,212 | 77 | 592 | 366 | 129 | 34 | 11 | 3 | - |  |
| **LVH-ECG, n (%)** |  |  |  |  |  |  |  |  |  |  |
| Absent | 799 (68·9) | 68 (90·7) | 431 (75·7) | 225 (64·8) | 56 (45·5) | 17 (53·1) | 1 (10·0) | 1 (33·3) | - |  |
| All LVH-ECG | 360 (31·1) | 7 (9·3) | 138 (24·3) | 122 (35·2) | 67 (54·5) | 15 (46·9) | 9 (90·0) | 2 (66·7) | <0·001 |  |
| LVH-ECG without strain | 297 (25·6) | 5 (6·7) | 125 (22·0) | 106 (30·5) | 47 (38·2) | 8 (25·0) | 5 (50·0) | 1 (33·3) | - |  |
| LVH-ECG with strain | 63 (5·4) | 2 (2·7) | 13 (2·3) | 16 (4·6) | 20 (16·3) | 7 (21·9) | 4 (40·0) | 1 (33·3) | - |  |
| Missing | 53 | 2 | 23 | 19 | 6 | 2 | 1 | 0 | - |  |
| **LVH-Echo, n (%)** | 1040 (90·0) | 69 (95·8) | 536 (94·7) | 313 (88·9) | 91 (75·8) | 24 (75·0) | 7 (63·6) | 0 (0·0) | <0·001 |  |
| Missing | 57 | 5 | 26 | 14 | 9 | 2 | 0 | 1 | - |  |
| **Global Longitudinal Strain, n (%)** |  |  |  |  |  |  |  |  |  |  |
| Normal | 662 (59·6) | 55 (76·4) | 391 (71·9) | 172 (51·3) | 33 (28·7) | 11 (34·4) | 0 (0·0) | 0 (0·0) | - |  |
| Borderline | 263 (23·7) | 10 (13·9) | 108 (19·9) | 98 (29·3) | 36 (31·3) | 8 (25·0) | 3 (27·3) | 0 (0·0) | - |  |
| Reduced | 186 (16·7) | 7 (9·7) | 45 (8·3) | 65 (19·4) | 46 (40·0) | 13 (40·6) | 8 (72·7) | 2 (100·0) | <0·001 |  |
| Missing | 101 | 5 | 48 | 31 | 14 | 2 | 0 | 1 | - |  |
| **LV Systolic Dysfunction, n (%)** |  |  |  |  |  |  |  |  |  |  |
| Normal | 999 (90·2) | 112 (99·1) | 586 (93·0) | 999 (90·2) | 112 (99·1) | 586 (93·0) | 301 (82·5) | 301 (82·5) | - |  |
| Mildly Reduced or Reduced | 109 (9·8) | 1 (1·4) | 31 (5·7) | 40 (11·8) | 25 (22·1) | 7 (22·6) | 4 (36·4) | 1 (100·0) | <0·001 |  |
| Missing | 104 | 6 | 49 | 28 | 16 | 3 | 0 | 2 | - |  |
| **Arterial Stiffness (n, %)** | 369 (38·0) | 15 (23·8) | 136 (29·9) | 133 (43·9) | 65 (59·1) | 12 (41·4) | 7 (77·8) | 1 (33·3) | <0·001 |  |
| Missing | 240 | 14 | 137 | 63 | 19 | 5 | 2 | 0 | - |  |
| **Renal impairment (n, %)**· | 44 (3·8) | 4 (5·3) | 8 (1·4) | 16 (4·7) | 14 (11·5) | 1 (3·1) | 0 (0·0) | 1 (33·3) | <0·001 |  |
| Missing | 66 | 2 | 32 | 22 | 7 | 2 | 1 | 0 | - |  |
| **Any TOD, n (%)** | 696 (57·4) | 31 (40·3) | 272 (45·9) | 240 (65·6) | 114 (88·4) | 25 (73·5) | 11 (100·0) | 3 (100·0) | <0·001 |  |
