# Supplementary material for: Prevalence of target organ damage in rural Kenya and The Gambia: a cross-sectional study
Source: Lancet Reg Health Afr. 2026 Sep;7:None. doi: 10.1016/j.lanafr.2026.100087 (PMC13561962; doi:10.1016/j.lanafr.2026.100087)
Supplement: Supplementary file 27 [file mmc9.docx]

Table S9: Continuous target organ damage variables stratified by blood pressure category. Results reported as mean and standard deviation. P-values are from analysis of variance between strata. Blood pressure categories defined using the 2024 European Society of Cardiology categories based on 24-hour ambulatory blood pressure measurement.

|  | **Overall** | **Non-elevated blood pressure** | **Elevated blood pressure** | **Hypertension** | **p-value** |  |
| --- | --- | --- | --- | --- | --- | --- |
| n | 1,212 | 120 | 686 | 406 | - |  |
| LV mass, g (mean, SD) | 118·7 (41·6) | 106·9 (33·8) | 111·3 (38·5) | 134·8 (44·1) | <0·001 |  |
| Indexed LV mass to allometric growth rate, g/m^2·7^ (mean, SD) | 33·5 (11·8) | 31·5 (9·5) | 31·2 (11·0) | 37·8 (12·6) | <0·001 |  |
| Indexed LV mass to body surface area, g/m^2^ (mean, SD) | 74·2 (24·1) | 71·0 (20·1) | 69·9 (22·5) | 82·4 (25·6) | <0·001 |  |
| Global longitudinal strain, % (mean, SD) | -18·6 (2·7) | -20·3 (2·2) | -19·1 (2·4) | -17·2 (2·8) | <0·001 |  |
| Left ventricular ejection fraction, % (mean, SD) | 56·8 (6·3) | 59·4 (4·8) | 57·5 (6·0) | 54·8 (6·8) | <0·001 |  |
| Pulse wave velocity, m/s (mean, SD) | 9·7 (1·6) | 9·0 (1·6) | 9·5 (1·5) | 10·1 (1·6) | <0·001 |  |
| Estimated glomerular filtration rate, mL/min/1·73m^2^ (mean, SD) | 96·4 (17·6) | 97·5 (17·0) | 98·9 (16·4) | 91·7 (18·9) | <0·001 |  |
